# Nomogram using intratumoral and peritumoral radiomics for the preoperative prediction of visceral pleural invasion in clinical stage IA lung adenocarcinoma

**DOI:** 10.1186/s13019-024-02807-7

**Published:** 2024-05-31

**Authors:** Yun Wang, Deng Lyu, Su Hu, Yanqing Ma, Shaofeng Duan, Yayuan Geng, Taohu Zhou, Wenting Tu, Yi Xiao, Li Fan, Shiyuan Liu

**Affiliations:** 1 Department of Radiology, Second Affiliated Hospital of Navy Medical University, 415 Fengyang Road, Huangpu District, Shanghai, 200003 China; 2 Department of Radiology, The First Affiliated Hospital of Soochow University, Suzhou, Jiangsu China; 3 Department of Radiology, Zhejiang Provincial People’s Hospital, Affiliated People’s Hospital of Hangzhou Medical College, Hangzhou, Zhejiang China; 4 GE Healthcare, Precision Health Institution, Shanghai, China; 5 Shukun(Beijing) Network Technology Co.,Ltd, Beijing, China

**Keywords:** Lung cancer, Adenocarcinoma, Visceral pleural invasion, Radiomics, Nomogram

## Abstract

**Background:**

Accurate prediction of visceral pleural invasion (VPI) in lung adenocarcinoma before operation can provide guidance and help for surgical operation and postoperative treatment. We investigate the value of intratumoral and peritumoral radiomics nomograms for preoperatively predicting the status of VPI in patients diagnosed with clinical stage IA lung adenocarcinoma.

**Methods:**

A total of 404 patients from our hospital were randomly assigned to a training set (*n* = 283) and an internal validation set (*n* = 121) using a 7:3 ratio, while 81 patients from two other hospitals constituted the external validation set. We extracted 1218 CT-based radiomics features from the gross tumor volume (GTV) as well as the gross peritumoral tumor volume (GPTV_5_, _10_, _15_), respectively, and constructed radiomic models. Additionally, we developed a nomogram based on relevant CT features and the radscore derived from the optimal radiomics model.

**Results:**

The GPTV_10_ radiomics model exhibited superior predictive performance compared to GTV, GPTV_5_, and GPTV_15_, with area under the curve (AUC) values of 0.855, 0.842, and 0.842 in the three respective sets. In the clinical model, the solid component size, pleural indentation, solid attachment, and vascular convergence sign were identified as independent risk factors among the CT features. The predictive performance of the nomogram, which incorporated relevant CT features and the GPTV_10_-radscore, outperformed both the radiomics model and clinical model alone, with AUC values of 0.894, 0.828, and 0.876 in the three respective sets.

**Conclusions:**

The nomogram, integrating radiomics features and CT morphological features, exhibits good performance in predicting VPI status in lung adenocarcinoma.

**Supplementary Information:**

The online version contains supplementary material available at 10.1186/s13019-024-02807-7.

## Background

Lung cancer ranks among the most prevalent and lethal malignant tumors [1], with lung adenocarcinoma (LUAD) constituting the predominant pathological subtype [2]. Visceral pleural invasion (VPI) is strongly related to adverse postoperative prognoses [3]. The presence of VPI elevates the T stage of lung cancer from T1 to T2 and advances the TNM stage from IA to IB [3]. Notably, recent research underscores the significant influence of VPI status on the decision-making process of thoracic surgeons regarding the selection of an appropriate surgical approach for lung cancer [4]. In cases where VPI is absent, sublobectomy serves as a viable option to preserve lung function.^3^ Conversely, if the tumor does invade the visceral pleura, a lobectomy coupled with a more extensive lymph node dissection may yield a more favorable prognosis [5]. Unfortunately, the conventional intraoperative diagnosis of VPI is time-consuming and prone to inaccuracies, with a reported accuracy of only 56.5% [6]. Consequently, the current gold standard, relying on postoperative pathological diagnosis [2], introduces a delay in determining an appropriate surgical plan, highlighting the need for a reliable preoperative predictive method for VPI status to ensure precise treatment of lung cancer.

Previous studies have used CT features to predict VPI status [7–16]. Notably, lung cancer presenting as a pure ground-glass nodule (pGGN) on CT or with no contact with the pleura was associated with a VPI-negative status [12–15]. Conversely, direct tumor-pleural contact, pleural indentation sign, and pleural tags were identified as high-risk CT features for predicting VPI status in LUAD [16]. However, the accuracy of VPI prediction based on different combinations of these high-risk CT signs ranged from 62.7 to 72.3%, with positive predictive values ranging from 44.1 to 56.4% [16]. This suggests a substantial proportion of false positive predictions, highlighting the ongoing challenge in accurately determining whether a part-solid or solid nodule in contact with the pleura has invaded the visceral pleura based on CT morphological features alone.

Radiomics, a method for extracting high-dimensional features from segmented images, including gray level changes and voxel spatial relationships, holds huge promise in achieving accurate diagnoses and prognosis assessments of diseases through feature selection and model establishment [17–19]. By leveraging radiomics, the subjective interpretation of CT morphological features by observers can be circumvented, and a wealth of digital information within the image, imperceptible to the human eye, can be comprehensively mined and integrated [20]. Previous pathological studies have indicated the presence of tumor-infiltrating lymphocytes and tumor-associated macrophages on the edge of invasive lesions [21], demonstrating associations with metastasis [22]. The peritumoral region, as an indicator of the tumor microenvironment, holds critical biological significance in reflecting the aggressive behavior of the tumor [23, 24]. Recent studies have demonstrated that incorporating radiomics features from the peritumoral region into modeling analysis can enhance predictive accuracy in the preoperative assessment of pathological invasiveness [17], lymphovascular invasion [18], lymph node metastasis [25], and spread through air space in lung cancer patients, compared to models relying solely on intratumoral features [26]. While previous investigations have successfully applied radiomics for assessing VPI status in early lung cancer [27–32], their primary focus has been on intratumoral features, with limited exploration of the potential contribution of peritumoral radiomics features. Additionally, the reliability and reproducibility of these models have not been validated in external sets.

In this study, we hypothesized that high-throughput feature extraction within the volume of interest (VOI), encompassing both intratumoral and peritumoral regions, can not only capture the tumor’s intrinsic heterogeneity but also comprehensively depict the spatial structural characteristics of the tumor and its adjacent tissues in a higher-dimensional space. Consequently, we developed a radiomics model based on the GTV and the incorporation of peritumoral regions at 5 mm, 10 mm, and 15 mm distances. Our objective was to assess the radiomics model’s effectiveness in predicting VPI status in early subpleural LUAD and to investigate whether its diagnostic performance could be further enhanced when combined with traditional CT morphological features.

## Methods

### Patients

A total of 1146 patients with clinical stage IA LUAD, who underwent surgery between July 2014 and July 2022 at three hospitals, were included retrospectively.

Inclusion criteria were as follows: (1) Clinical stage IA LUAD (cT1N0M0, with the maximum tumor length ≤ 3 cm); (2) Tumors located under the pleura, in direct contact with the pleura or connected to the pleura via lines or strips (pleural tags) [9]; (3) Minimum distance from the lesion to the pleura (DLP) ≤ 10 mm; (4) Thin-slice chest CT (slice thickness ≤ 2 mm) performed within 2 weeks before surgery; (5) Complete pathological report on VPI status.

Exclusion criteria were as follows: (1) Pathologically confirmed atypical adenomatous hyperplasia, or adenocarcinoma in situ (*n* = 71); (2) Tumors neither in direct contact with the pleural surface nor with pleural tags (*n* = 278); (3) DLP > 10 mm (*n* = 65); (4) Prior treatment or biopsy before CT (*n* = 15); (5) Time interval of more than 2 weeks between CT examination and surgery (*n* = 10); (6) Poor CT image quality (*n* = 78); (7) Presence of pGGN (*n* = 123); (8) Unavailability of VPI status pathological report (*n* = 21).

**Fig. 1:**
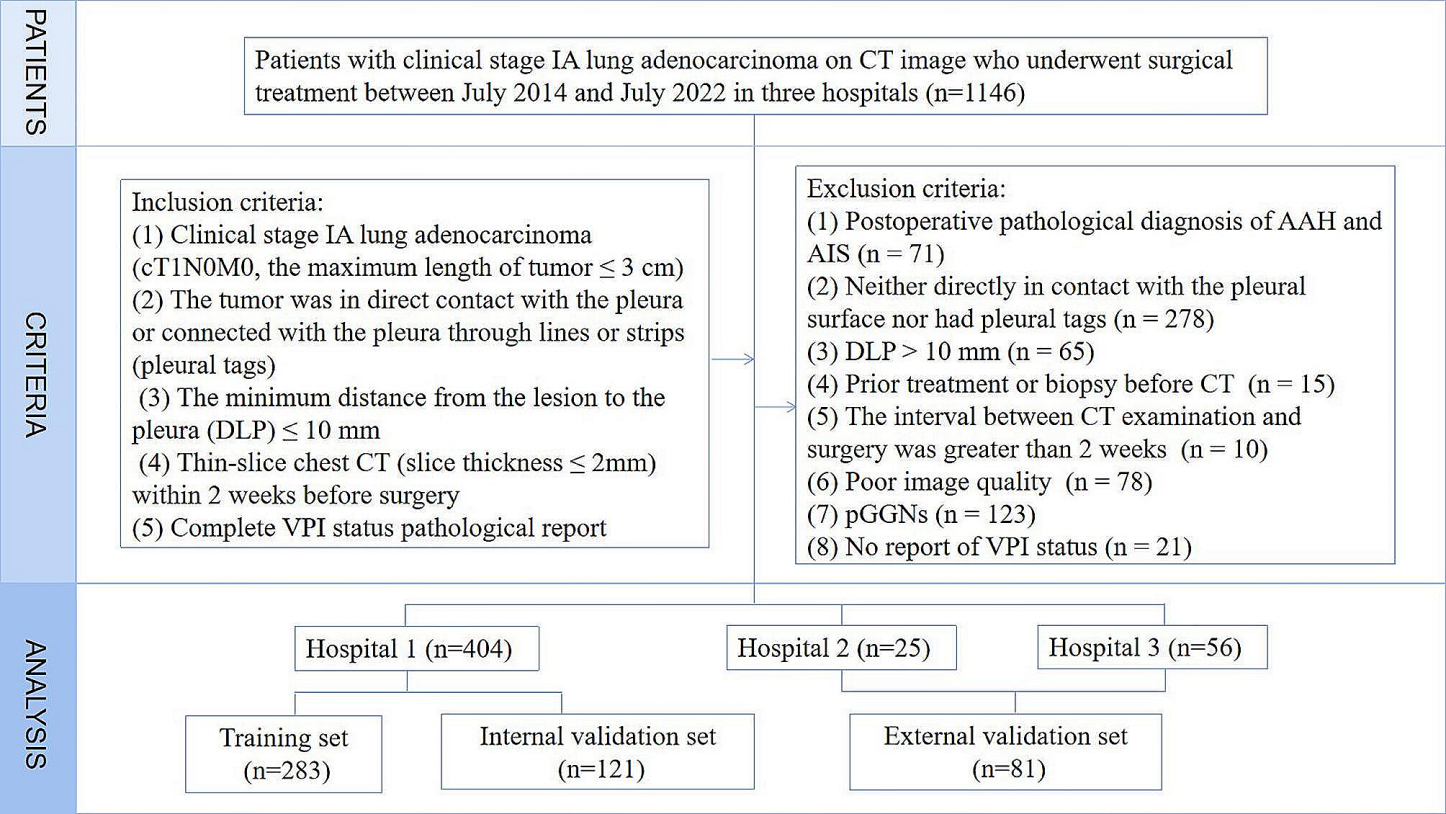
The flowchart of inclusion and exclusion criteria of patients

Ultimately, 404 patients from our institution (Hospital 1) were included as the internal cohort and were randomly divided into a training set (*n* = 283) and an internal validation set (*n* = 121) at a ratio of 7:3. An external validation set was formed, comprising patients from Hospital 2 (*n* = 25) and Hospital 3 (*n* = 56) (Fig. 1). Patients were categorized into two groups based on the VPI status determined during pathology: VPI-positive and VPI-negative. Specific details about the collected clinical and pathological data can be found in the Additional file 1. This study was approved by the Ethics Committee of our Hospital (decision number: CZ-20210528-01), and subjects’ informed consent was exempted.

### CT morphological features evaluation and clinical model construction

**Fig. 2:**
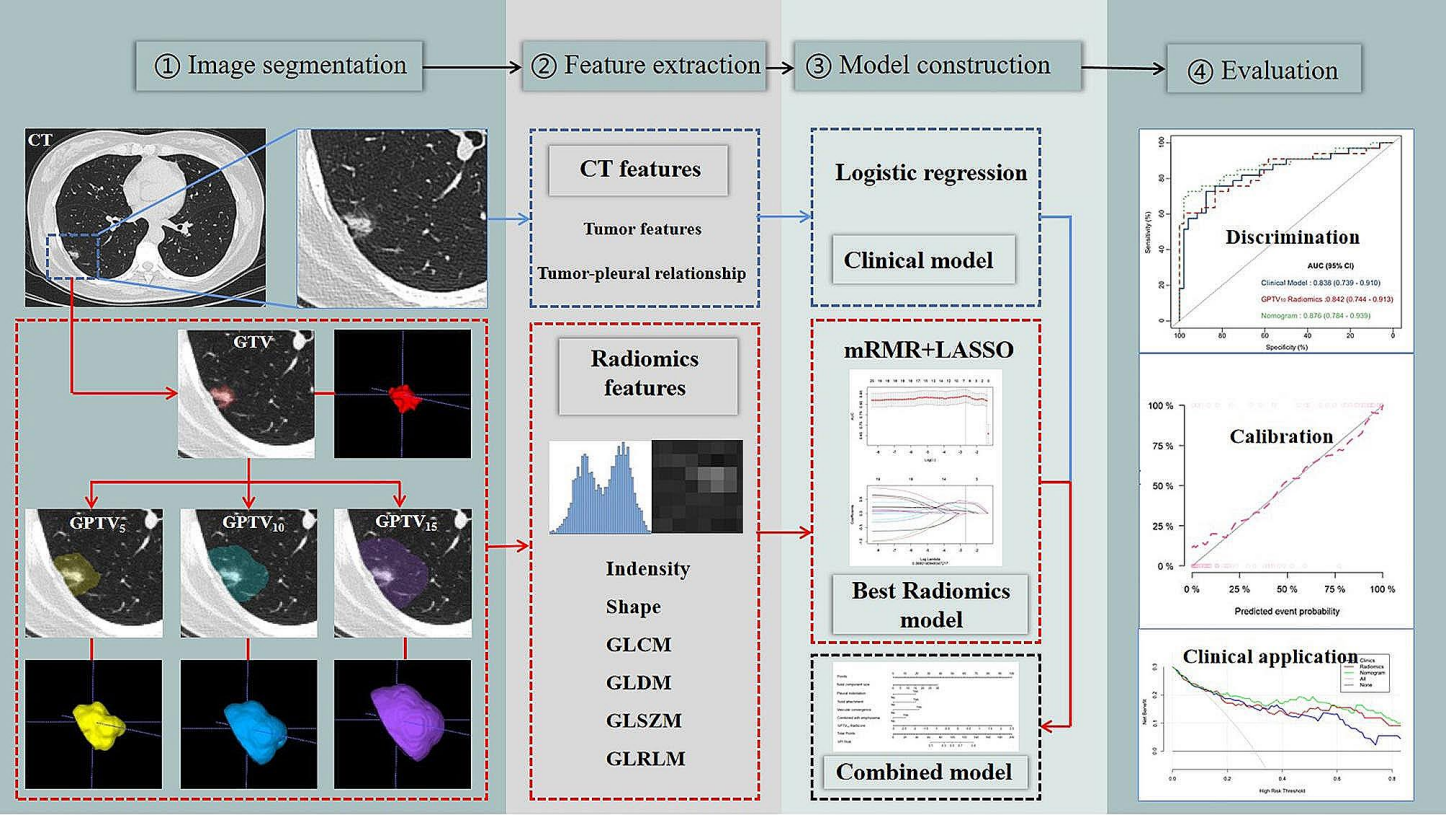
The workflow of this study

The study’s workflow is depicted in Fig. 2. CT image acquisition details are provided in the Additional file 2. The DICOM images of non-enhanced chest CT scans were imported into the Radiant DICOM Viewer software (Version 4.2.1, Medixant, Poland) for visualization of tumor CT morphological features through multi-planar reconstruction (MPR) and maximum density projection (MIP). Two thoracic radiologists with 7 years and 9 years of working experience blinded to patient information, conducted qualitative assessments and quantitative measurements of the tumors. Consensus was reached through consultation in cases of differing opinions regarding qualitative indicators. The average of the measurements by the two radiologists was used for quantitative parameters analysis.

The assessment included the following CT qualitative indicators: (i) Tumor-pleura signs, such as tumors in direct contact with the pleura (pleural attachment) or connected to the pleura via linear strands (pleural tags) [9] (ii) Pleura signs involving tumor-induced deviation of the pleura from its original position (pleural indentation) [15]; and the presence of a solid component of the tumor in direct contact with the pleura (solid attachment); (iii) Tumor signs, encompassing density type (solid or part-solid), shape (irregular or oval/round), interface (ill-defined or well-defined), two marginal characteristics (lobulation and spiculation), two internal features (vacuole sign, cavity or cyst sign), and three adjacent structural features (vascular convergence sign, air bronchogram signs, and emphysema in the lobe of the tumor) [33, 34].

**Fig. 3:**
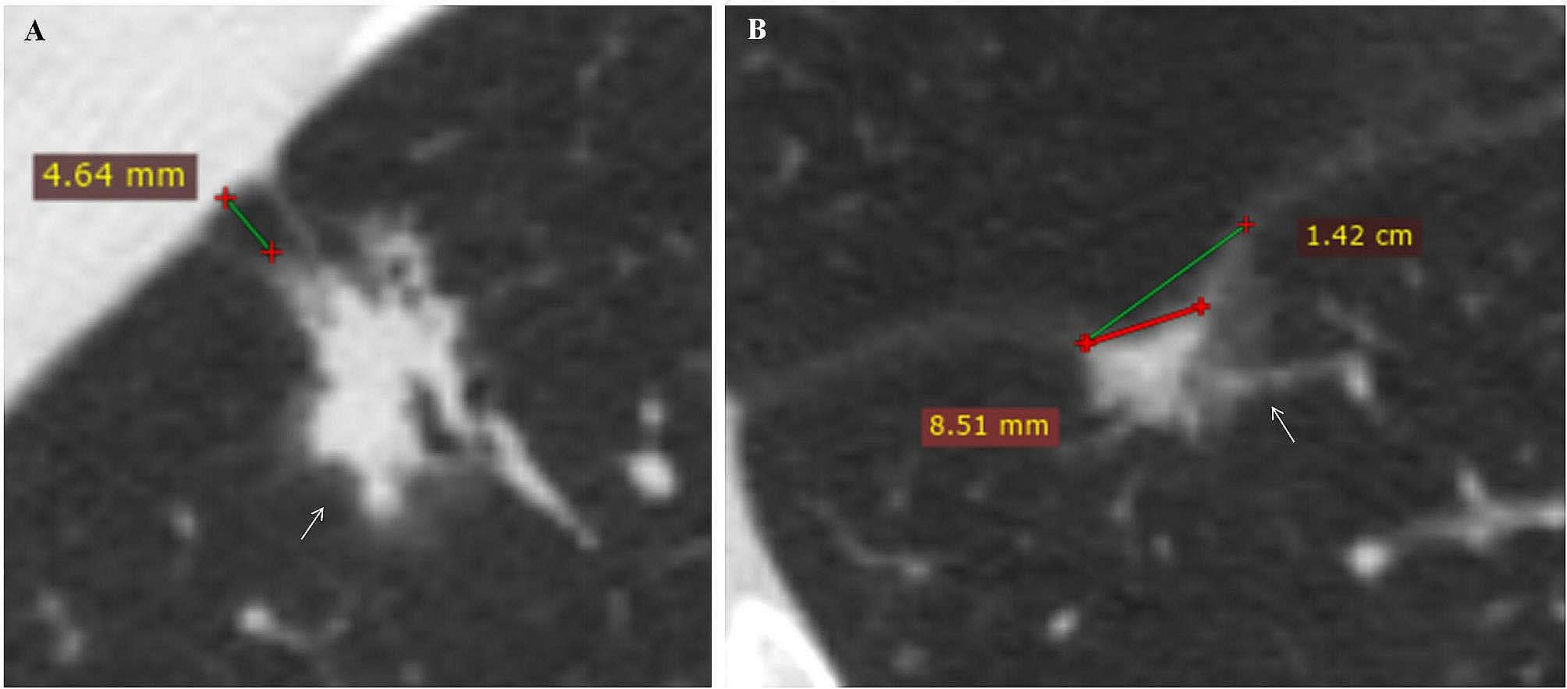
The illustration of the measurements of DLP, pleural contact length and solid pleural contact length. (**A**) A 53-year-old man presented with a mGGN in the upper lobe of the right lung (white arrow), with a DLP of 4.64 mm. The pathological results were invasive LUAD with VPI-positive. (**B**) A 64-year-old man presented with a mGGN in the lower lobe of the right lung (white arrow), which was in direct contact with the interlobar fissure and accompanied by pleural indentation sign, with solid components in contact with the pleura. The green line was a straight line to measure the pleura contact length (14.2 mm), and the red line was the solid pleural contact length (8.51 mm). The pathological results were invasive LUAD with VPI-positive.

For CT quantitative parameter measurement: (i) The maximum diameter of the tumor (tumor size, T) and the maximum diameter of the solid component (consolidation size, C) were measured on MPR lung window images, and the consolidation-tumor ratio (CTR) was calculated [35]. (ii) For tumors with pleural tags, the minimum vertical DLP at the lung window on MPR images was measured [7]. (iii) For tumors with pleural attachment and a DLP of 0 mm, the longest interface length of the entire tumor and solid component was measured using a straight line on the lung window of the MPR images [11]. The illustration of the measurements of DLP, pleural contact length and solid pleural contact length are shown in Fig. 3.

### CT images preprocessing and tumor segmentation

Prior to segmentation, standard resampling and grayscale discretization of CT images were performed. The ITK-SNAP software (version 3.80, www.itksnap.org) was used for slice-by-slice delineation of the GTV along the tumor boundary, with the 3D region defined as the VOI. GTV was defined as the entire visible tumor area identified subjectively by the radiologists. The segmentation aimed to exclude vessels, bronchi, and pleura as much as possible during the process.

The intraclass correlation coefficients (ICCs) were calculated to assess agreements between and within observers for the selection of radiomics features with high reproducibility and reliability. Inter-observer agreements were evaluated by two thoracic radiologists with 7 years and 9 years of working experience, who independently segmented 30 randomly selected tumors. Intra-observer agreements were assessed by the 9-year experienced radiologist, who re-segmented 30 tumors one month later. The remaining tumor segmentations were performed solely by the 9-year experienced radiologist, with both radiologists blinded to the patients’ pathological information during the segmentation process.

The VOI segmentation of the GTV was expanded outward by 5 mm, 10 mm, and 15 mm, respectively, and pixel filtering was conducted to automatically exclude peritumoral non-pulmonary tissues (such as peritumoral vessels, soft tissues, and chest wall, mediastinum, and abdominal structures) based on pixel thresholding using a Python platform (version 3.11). The resulting VOI segmentation of the GPTV in Nifty format was generated, named GPTV_5_, GPTV_10_, and GPTV_15_, accordingly.

### Radiomics feature extraction and data preprocessing

The extraction of features was carried out using Pyradiomics (version 3.0.1, https://pyradiomics.Read the docs.io/en/latest/changes.html). A total of 1218 radiomics features were extracted, with detailed feature types provided in Additional file 3 and 4. Given the inclusion of CT images from multiple hospitals and diverse CT protocols, the intensities of all radiomics features were normalized using the ComBat compensation method (https://forlhac.shinyapps.io/Shiny_ComBat) and z-score transformation (z = x-µ/σ) [36–38].

### Radiomics feature selection and model construction

ICCs were employed to evaluate the consistency between the segmented GTV and the corresponding GPTV_5_, GPTV_10_, and GPTV_15_ radiomics features for intra-observer and inter-observer assessments. The “psych” package of R software was used to assess the consistency of the radiomics features. To avoid overfitting, the maximal redundancy minimal relevance (mRMR) algorithm and the least absolute shrinkage and selection operator (LASSO) logistic regression method were applied to features with good consistency (ICC > 0.75) in the training set to limit the dimension of the features. A 10-fold cross-validation process was employed to select the optimal hyperparameter λ. Features with coefficients not equal to zero under the optimal λ were chosen for constructing the radiomics model. The radscore was calculated by summing the selected features, weighted by their coefficients. Wilcoxon tests were used to compare differences between the VPI-positive and VPI-negative groups. Radiomics models were developed based on GTV, GPTV_5_, GPTV_10_, and GPTV_15_, and their diagnostic efficacy was evaluated. The best radiomics model was determined by the highest AUC value in the validation set. The analysis process is depicted in Fig. 2.

### Clinical model and nomogram construction

In the training set, a multivariate logistic regression analysis was performed using clinical and CT morphological features with *P* < 0.1 in univariate logistic analysis. The best variable combinations were selected through a backward stepwise selection process to establish the clinical prediction model. The variables in the clinical model and the radscore from the optimal radiomics model were used as predictive variables to create the combined prediction model and the corresponding nomogram. The predictive performance of each model was assessed in both internal and external validation sets. The model development process is illustrated in Fig. 2.

### Statistical analysis

IBM SPSS Statistics (version 20.0, USA) and R software (version 4.2.2, http://www.Rproject.org) were used for statistical analysis. Details of the statistical analysis process are presented in Additional file 5.

## Results

### Clinical prediction model

Additional file 6 provides the clinical and pathological data for the three respective sets. Regarding CT features, there was strong agreement between the measurements of the two observers (ICC 0.954–0.996), and qualitative evaluation indicators exhibited high consistency (Kappa 0.832-1.000). Additional inter-observer agreement results for each CT feature are detailed in Additional file 7. Univariate analysis revealed significant differences (*P* < 0.05) between the VPI-positive and VPI-negative groups for several CT features, including tumor size, solid component size, CTR, whole tumor pleural contact length, solid component pleural contact length, density type, pleural indentation sign, solid attachment sign, spiculation sign, vascular convergence sign, and the presence of emphysema. Notably, the VPI-positive group exhibited a higher prevalence of solid nodules, solid attachments, pleural indentation, spiculation, and vascular convergence signs, along with significantly larger tumors and solid components, longer CTR, and longer whole tumor and solid pleural contact lengths (all *P* < 0.05; Table 1).

**Table 1:** Univariate analysis of CT features of clinical stage IA lung adenocarcinoma

CT features	Training set (*n* = 283)			Internal validation set (*n* = 121)			External validation set (*n* = 81)		
	VPI-Negative ( *n* = 149)	VPI-Positive (*n* = 134)	*P*-value	VPI-Negative (*n* = 61)	VPI-Positive (*n* = 60)	*P*-value	VPI-Negative(*n* = 48)	VPI-Positive (*n* = 33)	*P*-value
Tumor size (mm)	18.4 (15.0, 22.7)	24.1 (19.5, 27.0)	<0.001	19.1 (14.4, 22.4)	24.5 (20.7, 27.8)	<0.001	19.1 (13.7, 23.4)	21.0 (19.4, 28.6)	0.004
Solid component size (mm)	9.2 (5.0, 14.3)	19.4 (13.8, 23.0)	<0.001	9.3 (6.4, 14.0)	19.1 (13.2, 24.0)	<0.001	10.1 (5.0, 15.5)	18.5 (11.6, 22.3)	<0.001
CTR (%)	49.0 (26.5, 72.0)	85.6 (66.1, 100.0)	<0.001	53.7 (33.1, 79.8)	87.9 (67.5, 100.0)	<0.001	55.3 (36.6, 81.1)	81.8 (62.5, 96.6)	0.002
Pleural contact length (mm)	6.3 (0.0, 13.3)	10.9 (0.0, 17.8)	0.009	7.6 (0.0, 12.7)	7.2 (0.0, 16.4)	0.835	0.0 (0.0, 12.7)	10.0 (0.0, 14.7)	0.067
Solid pleural contact length (mm)	0.0 (0.0, 4.9)	8.1 (0.0, 12.8)	< 0.001	0.0 (0.0, 5.7)	2.0 (0.0, 10.7)	0.097	0.0 (0.0, 2.7)	6.4 (0.0, 11.9)	< 0.001
DLP (mm)	0.0 (0.0, 2.6)	0.0 (0.0, 2.6)	0.447	0.0 (0.0, 2.6)	0.0 (0.0, 2.8)	0.386	1.3 (0.0, 2.3)	0.0 (0.0, 2.4)	0.187
Tumor-pleura relationship			0.227^a^			0.238^a^			0.095^a^
Pleural tags	65 (43.6%)	49 (36.6%)		23 (37.7%)	29 (48.3%)		25 (52.1%)	11 (33.3%)	
Pleural attachment	84 (56.4%)	85 (63.4%)		38 (62.3%)	31 (51.7%)		23 (47.9%)	22 (66.7%)	
Pleural indentation			<0.001^a^			0.020^a^			0.002^a^
No	66 (44.3%)	24 (17.9%)		24 (39.3%)	12 (20.0%)		30 (62.5%)	9 (27.3%)	
Yes	83 (55.7%)	110 (82.1%)		37 (60.7%)	48 (80.0%)		18 (37.5%)	24 (72.7%)	
Solid attachment			< 0.001^a^			0.928^a^			0.001^a^
No	92 (61.7%)	50 (37.3%)		31 (50.8%)	30 (50.0%)		35 (72.9%)	12 (36.4%)	
Yes	57 (38.3%)	84 (62.7%)		30 (49.2%)	30 (50.0%)		13 (27.1%)	21 (63.6%)	
Density type			<0.001^a^			0.001^a^			0.183^b^
Part-solid	136 (91.3%)	80 (59.7%)		54 (88.5%)	38 (63.3%)		44 (91.7%)	26 (78.8%)	
Solid	13 (8.7%)	54 (40.3%)		7 (11.5%)	22 (36.7%)		4 (8.3%)	7 (21.2%)	
Shape			0.228^a^			0.712			0.082^a^
Irregular	34 (22.8%)	39 (29.1%)		16 (26.2%)	14 (23.3%)		19 (39.6%)	7 (21.2%)	
Round/Oval	115 (77.2%)	95 (70.9%)		45 (73.8%)	46 (76.7%)		29 (60.4%)	26 (78.8%)	
Lobulation			0.633^a^			0.660^b^			0.614^b^
No	7 (4.7%)	8 (6.0%)		2 (3.3%)	4 (6.7%)		4 (8.3%)	1 (3.0%)	
Yes	142 (95.3%)	126 (94.0%)		59 (96.7%)	56 (93.3%)		44 (91.7%)	32 (97.0%)	
Spiculation			< 0.001^a^			0.001^a^			0.008^b^
No	136 (91.3%)	75 (56.0%)		56 (91.8%)	40 (66.7%)		48 (100.0%)	27 (81.8%)	
Yes	13 (8.7%)	59 (44.0%)		5 (8.2%)	20 (33.3%)		0 (0.0%)	6 (18.2%)	
Interface			0.223^c^			N/A			0.407^c^
Ill-defined	0 (0.0%)	2 (1.5%)		0 (0.0%)	0 (0.0%)		0 (0.0%)	1 (3.0%)	
Well-defined	149 (100.0%)	132 (98.5%)		61 (100.0%)	60 (100.0%)		48 (100.0%)	32 (97.0%)	
Air bronchogram			0.058^a^			0.169^a^			0.088^a^
No	89 (59.7%)	65 (48.5%)		32 (52.5%)	24 (40.0%)		35 (72.9%)	18 (54.5%)	
Yes	60 (40.3%)	69 (51.5%)		29 (47.5%)	36 (60.0%)		13 (27.1%)	15 (45.5%)	
Vacuole sign			0.080^a^			0.478^a^			0.260^a^
No	115 (77.2%)	91 (67.9%)		39 (63.9%)	42 (70.0%)		34 (70.8%)	27 (81.8%)	
Yes	34 (22.8%)	43 (32.1%)		22 (36.1%)	18 (30.0%)		14 (29.2%)	6 (18.2%)	
Cavity or cyst sigh			0.482^a^			0.774^a^			0.415^b^
No	139 (93.3%)	122 (91.0%)		55 (90.2%)	55 (91.7%)		43 (89.6%)	32 (97.0%)	
Yes	10 (6.7%)	12 (9.0%)		6 (9.8%)	5 (8.3%)		5 (10.4%)	1 (3.0%)	
Vascular convergence sign			< 0.001^a^			0.064^a^			0.014^b^
No	144 (96.6%)	103 (76.9%)		58 (95.1%)	51 (85.0%)		47 (97.9%)	26 (78.8%)	
Yes	5 (3.4%)	31 (23.1%)		3 (4.9%)	9 (15.0%)		1 (2.1%)	7 (21.2%)	
Emphysema			< 0.001^a^			0.721^b^			0.019^b^
No	146 (98.0%)	117 (87.3%)		57 (93.4%)	54 (90.0%)		46 (95.8%)	25 (75.8%)	
Yes	3 (2.0%)	17 (12.7%)		4 (6.6%)	6 (10.0%)		2 (4.2%)	8 (24.2%)	

*Note *^a^: Pearson’s chi-squared test. ^b^: Yate’s correction for continuity. ^c^: Fisher’s exact test

VPI, visceral pleural invasion; CTR, consolidation-to-tumor ratio; DLP, minimum distance between lesion and pleura

Multivariate logistic regression analysis was employed to select the optimal combination of predictive variables for constructing the clinical model. The independent risk factors for VPI were the solid component size (OR = 1.23, 95% CI 1.16 ~ 1.30, *P* < 0.001), pleural indentation sign (OR = 3.36, 95% CI 1.61 ~ 7.02, *P* = 0.001), solid attachment sign (OR = 2.98, 95% CI 1.56 ~ 5.70, *P* < 0.001), and vascular convergence sign (OR = 4.51, 95% CI 1.49 ~ 13.69, *P* = 0.008), as shown in Table 2.

**Table 2:** Univariate and multivariate logistic regression analysis of the CT features in the training set

	Univariate Logistic Regression			Multivariate Logistic Regression	
	OR (95% CI)	*P* value		OR (95% CI)	*P* value
Tumor size	1.17 (1.11–1.23)	< 0.001			
Solid component size	1.25 (1.19–1.31)	< 0.001		1.23 (1.16–1.30)	< 0.001
CTR	1.05 (1.04–1.06)	< 0.001			
Pleural contact length	1.04 (1.01–1.07)	0.005			
Solid pleural contact length	1.14 (1.09–1.19)	< 0.001			
Density type	7.06 (3.63–13.74)	< 0.001			
Pleural indentation	3.64 (2.11–6.30)	< 0.001		3.36 (1.61–7.02)	0.001
Solid attachment	2.71 (1.68–4.39)	< 0.001		2.98 (1.56–5.70)	< 0.001
Spiculation	8.23 (4.24–15.98)	< 0.001			
Air bronchogram	1.57 (0.98–2.52)	0.059			
Vascular convergence	8.67 (3.26–23.05)	< 0.001		4.51 (1.49–13.69)	0.008
Combined with emphysema	7.07 (2.02–24.71)	0.002		2.92 (0.68–12.63)	0.151

*Note* CTR, consolidation-to-tumor ratio; OR, odds ratio; CI, confidence interval

### Radiomics model

**Fig. 4:**
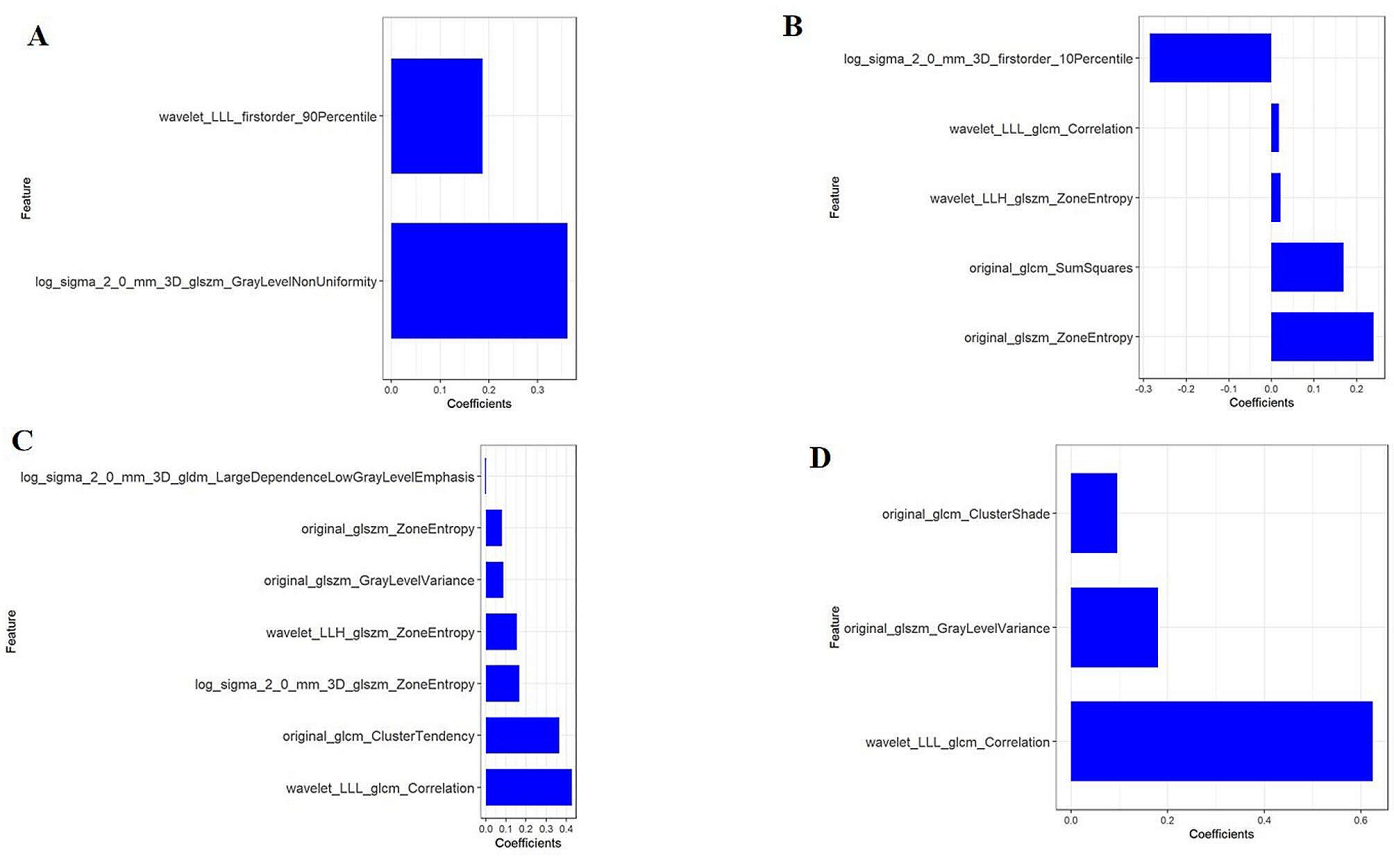
Retained radiomics features and corresponding coefficients of different models after dimensionality reduction by LASSO regression analysis (**A**) GTV model, (**B**) GPTV_5_ model, (**C**) GPTV_10_ model, (**D**) GPTV_15_ model

A total of 1218 radiomic features were extracted from the VOI of GTV, GPTV_5_, GPTV_10_, and GPTV_15_, respectively. Among these features, 72.2% (880/1218) of GTV features, 92.3% (1124/1218) of GPTV_5_ features, 97.1% (1183/1218) of GPTV_10_ features, and 97.8% (1191/1218) of GPTV_15_ features demonstrated good repeatability, with inter-class and intra-class ICCs exceeding 0.75. Among the features with ICC > 0.75, the mRMR algorithm was initially used to eliminate redundant and irrelevant features, retaining 30 features in each group. Subsequently, the LASSO regression algorithm was applied to select the optimized feature subset for constructing the final model. A 10-fold cross-validation process was used to determine the optimal hyperparameter λ. The optimal λ values for GTV, GPTV_5_, GPTV_10_, and GPTV_15_ were 0.153, 0.131, 0.068, and 0.100, respectively (see Additional file 8). With these optimal λ values, 2, 5, 7, and 3 features were selected to construct the radiomics models for GTV, GPTV_5_, GPTV_10_, and GPTV_15_, respectively (Fig. 4). The features used for model construction and their ICC details are provided in Additional file 9. The radscore formulas for the four radiomics models can be found in Additional file 10. The radscore for VPI-positive groups in all models was significantly higher than for VPI-negative groups (all *P* < 0.05), as shown in Additional file 11.

### Efficacy comparison of radiomics models

The AUC values for the GTV, GPTV_5_, GPTV_10_, and GPTV_15_ models in the training set for predicting VPI status were 0.838, 0.849, 0.855, and 0.841, respectively. In the internal validation set, the corresponding AUC values were 0.808, 0.855, 0.842, and 0.824. Similarly, in the external validation set, the AUC values were 0.809, 0.826, 0.842, and 0.823, respectively. The prediction performance of each radiomics model is summarized in Table 3, and the ROC curves for each radiomics model in the three sets can be found in Additional file 12. The DeLong test indicated that in the training set, the GPTV_10_ model outperformed the GPTV_15_ model, and the difference was statistically significant (Z = 2.076, *P* < 0.05). In the internal validation set, the performance of GPTV_5_ and GPTV_10_ models was superior to that of GTV, and the differences were statistically significant (Z = 3.030 and 2.163, both *P* < 0.05). The radiomics model with the highest AUC value in the external validation set was selected as the best radiomics model. Consequently, a combined model was constructed based on the GPTV_10_ model’s radscore and CT morphological features.

**Table 3:** Prediction performance of GTV, GPTV_5_, GPTV_10_, and GPTV_15_ radiomics models in three sets

Radiomics model	Set	AUC (95%CI)	Cut-off	Accuracy (%)	Sensitivity (%)	Specificity (%)
GTV	Training	0.838 (0.790–0.879)	-0.154	77.39	78.36	76.51
	Internal validation	0.808 (0.726–0.874)		74.38	63.33	85.25
	External validation	0.809 (0.706–0.888)		82.72	60.61	97.92
GPTV_5_	Training	0.849 (0.801–0.888)	-0.338	76.33	88.06	65.77
	Internal validation	0.855 (0.779–0.912)		77.69	68.33	86.89
	External validation	0.826 (0.726–0.902)		82.72	63.64	95.83
GPTV_10_	Training	0.855 (0.808–0.894)	-0.204	76.33	78.36	74.50
	Internal validation	0.842 (0.764–0.902)		77.69	63.33	91.80
	External validation	0.842 (0.744–0.913)		82.72	60.61	97.92
GPTV_15_	Training	0.841 (0.794–0.882)	0.193	75.97	64.18	86.58
	Internal validation	0.824 (0.744–0.887)		76.86	66.67	86.89
	External validation	0.823 (0.723–0.899)		82.72	63.64	95.83

*Note* AUC, area under curve; CI, confidence interval

### Efficacy evaluation of combined models

The radscore from the GPTV_10_ model and CT morphological features included in the clinical model were used as predictive variables to construct a combined model and the corresponding nomogram. The formula for the combined model was as follows:

Nomoscore = (Intercept) * -2.738 + solid component size * 0.076 + pleural indentation sign * 1.169 + the presence of solid component contact pleura * 1.178 + vascular convergence sign * 1.329 + the presence of combined emphysema * 0.650 + GPTV_10_-radscore * 1.110.

**Fig. 5:**
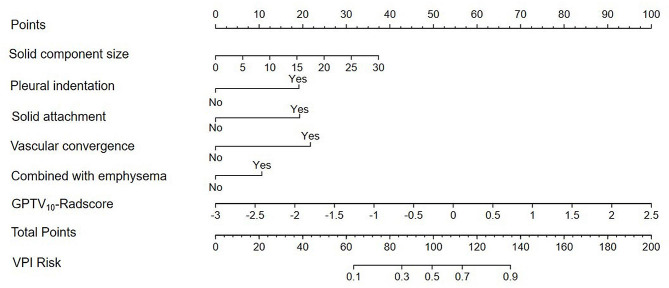
The developed nomogram based on GPTV_10_-Radscore and CT morphological features for predicting VPI status in clinical stage IA LUAD patients

**Fig. 6:**
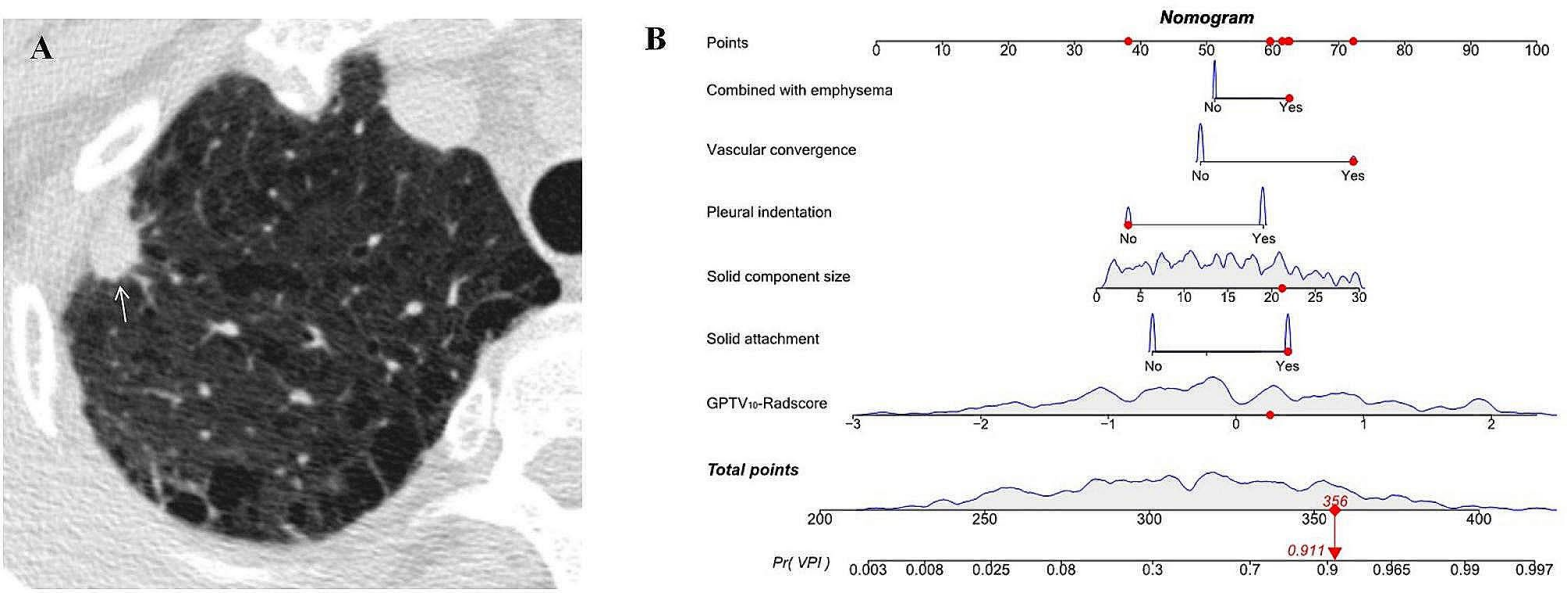
An example of the nomogram in clinical application. This was an axial non-enhanced chest CT image of a 64-year-old male patient with a solid nodule in the upper lobe of the right lung (white arrow) with invasive LUAD and VPI-positive. The factors in the nomogram were analyzed as follows: combined with emphysema = “Yes”, vascular convergence = “Yes”, pleural indentation = “No”, solid component size = 21.1 mm, solid attachment = ”Yes”, GPTV_10_-Radscore = 0.267, total score was 356 and the probability of VPI-positive was 0.911

**Fig. 7:**
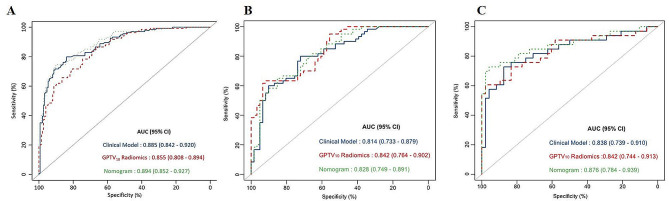
ROC curves of GPTV_10_ radiomics model, clinical model and combined model in three sets (**A**) training set, (**B**) internal validation set, (**C**) external validation set

The AUC values of the clinical model were 0.885, 0.814, and 0.838 in the three respective sets. The AUC values of the combined model were 0.894, 0.828, and 0.876 in the three respective sets, as presented in Table 4. The nomogram and examples of its clinical application can be found in Figs. 5 and 6. The ROC curves of the GPTV_10_-based radiomics model, clinical model, and combined model for predicting VPI in the three sets are shown in Fig. 7.

**Table 4:** Prediction performance of clinical model, GPTV_10_-radiomics model and combined model in three sets

Model	Set	Cut-off	AUC (95%CI)	Accuracy (%)	Sensitivity (%)	Specificity (%)
Clinical model	Training	0.010	0.885 (0.842–0.920)	81.98	79.85	83.89
	Internal validation		0.814 (0.733–0.879)	76.03	80.00	72.13
	External validation		0.838 (0.739–0.910)	81.48	72.73	87.50
GPTV_10_-Radiomics	Training	-0.204	0.855 (0.808–0.894)	76.33	78.36	74.50
	Internal validation		0.842 (0.764–0.902)	77.69	63.66	91.80
	External validation		0.842 (0.744–0.913)	82.72	60.61	97.92
Combined model	Training	0.394	0.894 (0.852–0.927)	82.69	74.63	89.93
	Internal validation		0.828 (0.749–0.891)	75.21	65.00	85.25
	External validation		0.876 (0.784–0.939)	86.42	72.73	95.83

**Fig. 8:**
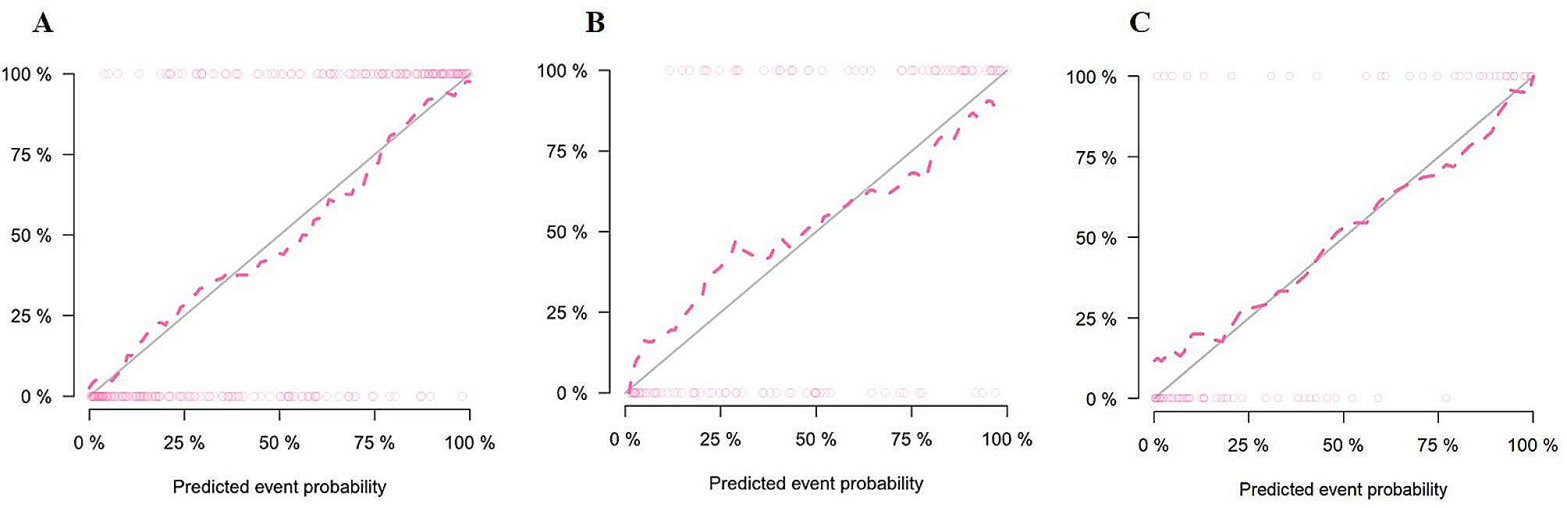
The calibration curves of the combined model in three sets (**A**) training set, (**B**) internal validation set, (**C**) external validation set

**Fig. 9:**
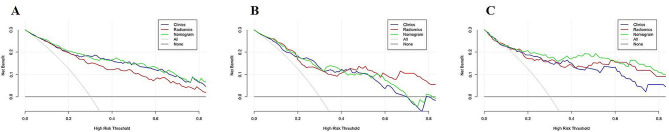
DCA for the nomogram, radiomics model, and clinical model in three sets. The y-axis represents the net benefit, and the x-axis represents the threshold probability. (**A**) training set, (**B**) internal validation set, (**C**) external validation set

The DeLong test showed that the combined model outperformed the GPTV_10_ radiomics model in the training set (Z = 2.987, *P* < 0.05). In the external validation set, the combined model performed better than the clinical model (Z = 2.348, *P* < 0.05). The Hosmer-Lemeshow test indicated that the combined model was a good fit in all three sets (all *P* > 0.05), as shown in Fig. 8. The decision curve analysis (DCA) curves revealed that the combined model achieved a better net benefit in predicting VPI status than the clinical model and the GPTV_10_ radiomics model, as shown in Fig. 9.

## Discussion

For part-solid or solid nodules in contact with the pleura, selecting the optimal surgical approach remains challenging for thoracic surgeons. In this respect, given that VPI is associated with a high risk of recurrence after sublobectomy [39, 40], it is essential to avoid misdiagnosing lung cancer with VPI. Moreover, in cases where patients have low pulmonary function or high surgical risk, the decision to opt for lobectomy should be made cautiously, especially when the tumor’s location allows for sublobectomy [41]. Therefore, accurately determining the presence of VPI is of great importance. Intraoperative frozen pathology for accurately diagnosing VPI status requires comprehensive histological sampling of the tumor and its adjacent pleura. However, this approach may extend the operation time and lead to unnecessary complications [40]. In this study, we developed a combined prediction model based on the GPTV_10_ radiomics model and traditional CT signs. In the external validation set, the combined model achieved an AUC value of 0.876, an accuracy of 86.42%, a sensitivity of 72.73%, and a high specificity of 95.83%. Furthermore, we constructed a nomogram that provides a visual representation of the intricate regression equation inherent in the combined model. The nomogram can streamline patient assessment and furnish thoracic surgeons with valuable insights for selecting the optimal surgical strategy.

The clinical model constructed in this study included five CT features: solid component size, solid attachment sign, pleural indentation sign, vascular convergence sign, and the presence of emphysema. The presence of a solid component is often indicative of the more aggressive nature of lung adenocarcinoma, serving as a criterion for T staging in clinical stage IA lung cancer [42]. Larger solid components correlate positively with tumor aggressiveness. Additionally, when a solid component is in contact with the pleura, the risk of visceral pleural invasion increases. As tumor malignancy increases, fibrous hyperplasia occurs within the tumor, leading to more significant traction on adjacent tissues. This traction results in morphological changes in nearby bronchi, vessels, and pleura, which manifest as air bronchogram signs, vascular convergence signs, and pleural indentation signs on CT images [43, 44]. Previous research has confirmed that LUAD accompanied by emphysema exhibits more aggressive characteristics and lower disease-free survival rates compared to cases without emphysema [45, 46] Consistently, our study indicated that LUAD with emphysema is more prone to invade the visceral pleura [47]. The solid component size reflects the invasiveness of the tumor itself, while the other features reflect the relationship between the tumor and the adjacent pleura, vessels, and pulmonary background. The clinical model, which incorporates both tumor and peritumoral CT features, provides a more comprehensive assessment than previous studies [16]. It exhibited promising predictive performance with AUC values of 0.885, 0.814, and 0.838 in the three respective sets.

In this study, precise GTV segmentation formed the basis for automatically expanding three different gradient ranges of the peritumoral region, thereby obtaining the integrated 3D segmentation of GPTV. This exploration marked the first attempt to identify the most efficient radiomics model for predicting VPI status in early LUAD, with a multi-center research providing an evaluation of the model’s generalizability. Emphasizing both the relationship between the tumor and adjacent visceral pleura and the tumor’s inherent aggressiveness, the peritumoral region was not solely utilized as VOI for model construction. The results demonstrated that the predictive efficacy of all GPTV radiomics models was superior to that of GTV, underscoring the peritumoral region’s ability to represent the tumor microenvironment and reflect tumor aggressiveness to a certain extent, as noted in previous studies [17, 23–26, 48]. In addition, the GPTV_10_-radiomics model exhibited superior performance compared to other models, likely due to the inclusion of tumors with a DLP ranging from 0 mm to 10 mm, where the 10 mm peritumoral extension range accurately covered various high-order features representing the three-dimensional spatial structure relationship between the tumor and pleura.

Building upon the GPTV_10_ VOI, seven optimal quantitative radiomics features were selected to indirectly reflect the biological differences between VPI-positive and VPI-negative patients. These features included Large Dependence Low Gray Level Emphasis (LDLGLE), Zone Entropy, Gray Level Variance, Cluster Tendency, and Correlation. LDLGLE, Zone Entropy, and Gray Level Variance are parameters of the Gray Level Size Zone Matrix (GLSZM), while Cluster Tendency and Correlation are parameters of the Gray Level Co-occurrence Matrix (GLCM), all of which are texture feature parameters [49]. It is well-established that GLCM reflects the spatial relationship between pixels, GLDM reflects the gray level relationship between the center pixel and its neighborhood, and GLSZM provides information about the uniformity of each gray level on a three-dimensional image [17]. The higher values of LDLGLE, regional entropy, gray variance, cluster trend, and correlation indicated a more uneven distribution of image texture and more irregular gray changes, signifying higher spatial heterogeneity of the tumor [18], and reflecting its strong invasiveness and increased risk of adjacent VPI.

The clinical model and radiomics model were found to be complementary in this study. Although their diagnostic performance was comparable, their integration exhibited superior diagnostic efficiency. The Delong test results indicated that the combined model outperformed the GPTV_10_ radiomics model in the training set (*P* < 0.05) and the clinical model in the external validation set (*P* < 0.05). The calibration curve demonstrated the model’s predicted probabilities to be in acceptable agreement with the actual probability, while the DCA illustrated that the combined model provided more net benefit than both the clinical model and the radiomics model.

There were several previous studies on predicting VPI status in lung adenocarcinoma by CT-based radiomics features [27–32]. Compared with previous studies, our study has some innovative points in patient enrollment and research methods. Firstly, lung tumors with specific VPI-negative on CT presentation such as pGGN or unrelated to the pleura were excluded in our study, therefore, our results were more objective in including cases with potential VPI-positive status to study the predictive performance of radiomics model combined with CT features. Secondly, our study explored the value of radiomics models based on GTV and GPTV with different peritumoral areas for predicting VPI status, no similar research has been reported before. Thirdly, based on the TRIPOD statement, different prediction models were established, and a multi-center dataset was included for internal and external validation of the model to verify the generalization of the models, which was lacking in previous studies.

While this study has notable strengths, it is not devoid of limitations. Firstly, the manual delineation of tumor segmentation was both time-consuming and labor-intensive; however, this method yielded a high level of segmentation accuracy. Moreover, the utilization of Python platform editing code for automatic peritumoral area acquisition, based on semi-automatic and manually precise tumor segmentation, significantly improved segmentation efficiency. The approach included the implementation of a threshold value to eliminate non-lung parenchyma areas, such as soft tissue of the chest wall, ribs, neck, mediastinum, and abdominal regions. This not only preserved the accuracy of artificial tumor delineation but also mitigated subjective errors in the manual delineation of the peritumoral area. Secondly, the sample size in this study was relatively small. Thirdly, the retrospective nature of the study inevitably resulted in selection bias. Additionally, some cases lacked clear PL1 and PL2 pathological grades, precluding the possibility of conducting subgroup analyses for these categories. Further studies are warranted in the future, with a focus on collecting a larger number of cases and more detailed information on pathological grading to address these limitations.

## Conclusions

In conclusion, radiomics features-based quantitative analysis provides a noninvasive and accurate diagnostic tool for reflecting the biological behavior of tumors. Combining radiomics features based on segmented GTPV_10_ with traditional CT morphological signs recognized by radiologists maximizes the diagnostic efficacy for preoperative prediction of VPI in clinical stage IA LUAD, thereby contributing to personalized and accurate treatment strategies.

### Electronic supplementary material

Below is the link to the electronic supplementary material.

Supplementary Material 1

## Data Availability

No datasets were generated or analysed during the current study.

## Abbreviations

VPI: Visceral pleural invasion
GTV: Gross tumor volume
GPTV: Gross peritumoral tumor volume
AUC: Area under the curve
LUAD: Lung adenocarcinoma
pGGN: Pure ground-glass nodule
VOI: Volume of interest
DLP: Distance from the lesion to the pleura
MPR: Multi-planar reconstruction
CTR: Consolidation-tumor ratio
ICCs: Intraclass correlation coefficients
mRMR: maximal redundancy minimal relevance
LASSO: The least absolute shrinkage and selection operator
ROC: Receiver operating characteristic
DCA: Decision curve analysis
