# Supplementary material for: Nomogram using intratumoral and peritumoral radiomics for the preoperative prediction of visceral pleural invasion in clinical stage IA lung adenocarcinoma
Source: J Cardiothorac Surg. 2024 May 31;19:307. doi: 10.1186/s13019-024-02807-7 (PMC11141037; doi:10.1186/s13019-024-02807-7)

**Supplementary Material**

**Additional file 1,** Clinical and pathological data collection

The clinical data of the patients were recorded, including age, gender, tumor location, type of surgery, and pathological diagnosis about histological subtype and VPI status.

The resected tissues were stained with hematoxylin and eosin, and the pathological diagnosis were performed by two pathologists with at least 10 years of experience. The specific elastic fiber stain was performed if the VPI status could not be determined accurately. Additionally, VPI was classified as no pleural invasion beyond the elastic layer (PL0), tumor invasion beyond the elastic layer (PL1), and tumor invasion to the surface of the visceral pleura (PL2), with PL1 and PL2 indicating the VPI-positive.

**Additional file 2,** CT image acquisition

Patients in the internal set (Hospital 1) underwent preoperative chest CT examination with four CT scanners, Toshiba Aquilion 16, GE Light Speed VCT, Philips Ingenuity 64 and Brilliance iCT, respectively.

In the external set, Siemens SOMATOM Definition AS and AS+ 64 row CT scanners and Philips Brilliance 16 were used in Hospital 2. Patients in the Hospital 3 underwent preoperative chest CT examination with Toshiba Aquilion 16, GE Revolution 256 and GE Discovery CT 750HD, Siemens SOMATOM Sensation 64 row and Definition Flash dual-source CT, Philips Brilliance 16 and iCT, Philips IQon Spectral CT.

The chest CT scanning parameters were as follows: tube voltage 120 kV, tube current 150 to 250 mAs or automatic tube current control; scanning layer thickness 5 mm; matrix 512 * 512; pitch 0.516 or 0.98 or 1 s; reconstruction layer thickness and layer interval 0.625mm, 1 mm, 1.5mm or 2mm; high and standard resolution algorithms; Non-contrast-enhanced images were used for this study.

**Additional file 3 Total 100 original radiomics features**

|  | Shape | First order | GLCM | GLRLM | GLSZM | GLDM |
| --- | --- | --- | --- | --- | --- | --- |
| 1 | Elongation | 10Percentile | Autocorrelation | GrayLevelNonUniformity | GrayLevelNonUniformity | DependenceEntropy |
| 2 | Flatness | 90Percentile | JointAverage | GrayLevelNonUniformityNormalized | GrayLevelNonUniformityNormalized | DependenceNonUniformity |
| 3 | LeastAxisLength | Energy | ClusterProminence | GrayLevelVariance | GrayLevelVariance | DependenceNonUniformityNormalized |
| 4 | MajorAxisLength | Entropy | ClusterShade | HighGrayLevelRunEmphasis | HighGrayLevelZoneEmphasis | DependenceVariance |
| 5 | Maximum2DDiameterColumn | InterquartileRange | ClusterTendency | LongRunEmphasis | LargeAreaEmphasis | GrayLevelNonUniformity |
| 6 | Maximum2DDiameterRow | Kurtosis | Contrast | LongRunHighGrayLevelEmphasis | LargeAreaHighGrayLevelEmphasis | GrayLevelVariance |
| 7 | Maximum2DDiameterSlice | Maximum | Correlation | LongRunLowGrayLevelEmphasis | LargeAreaLowGrayLevelEmphasis | HighGrayLevelEmphasis |
| 8 | Maximum3DDiameter | MeanAbsoluteDeviation | DifferenceAverage | LowGrayLevelRunEmphasis | LowGrayLevelZoneEmphasis | LargeDependenceEmphasis |
| 9 | MeshVolume | Mean | DifferenceEntropy | RunEntropy | SizeZoneNonUniformity | LargeDependenceHighGrayLevelEmphasis |
| 10 | MinorAxisLength | Median | DifferenceVariance | RunLengthNonUniformity | SizeZoneNonUniformityNormalized | LargeDependenceLowGrayLevelEmphasis |
| 11 | Sphericity | Minimum | JointEnergy | RunLengthNonUniformityNormalized | SmallAreaEmphasis | LowGrayLevelEmphasis |
| 12 | SurfaceArea | Range | JointEntropy | RunPercentage | SmallAreaHighGrayLevelEmphasis | SmallDependenceEmphasis |
| 13 | SurfaceVolumeRatio | RobustMeanAbsoluteDeviation | Imc1 | RunVariance | SmallAreaLowGrayLevelEmphasis | SmallDependenceHighGrayLevelEmphasis |
| 14 | VoxelVolume | RootMean  Squared | Imc2 | ShortRunEmphasis | ZoneEntropy | SmallDependenceLowGrayLevelEmphasis |
| 15 |  | Skewness | Idm | ShortRunHighGrayLevelEmphasis | ZonePercentage |  |
| 16 |  | TotalEnergy | Idmn | ShortRunLowGrayLevelEmphasis | ZoneVariance |  |
| 17 |  | Uniformity | Id |  |  |  |
| 18 |  | Variance | Idn |  |  |  |
| 19 |  |  | InverseVariance |  |  |  |
| 20 |  |  | MaximumProbability |  |  |  |
| 21 |  |  | SumEntropy |  |  |  |
| 22 |  |  | SumSquares |  |  |  |

**Additional file 4 Total 1218 radiomics features**

|  | Original | Laplacian of Gaussian filter | Wavelet |
| --- | --- | --- | --- |
| Shape (14) | 14×1 |  |  |
| First-order Statistics (18) | 18×1 | 18×5 | 18×8 |
| Texture (68) | 68×1 | 68×5 | 68×8 |

Reference**:** van Griethuysen JJ, Fedorov A, Parmar C, Hosny A, Aucoin N, Narayan V, Beets-Tan RG, Fillion-Robin J-C, Pieper S, Aerts HJJCr: Computational radiomics system to decode the radiographic phenotype. 2017;77:e104-e107

**Additional file 5,** Statistical analysis

The Shapiro-Wilk test was used to examine the normality of numeric variables. The normally distributed numerical variables were represented as the mean ± standard deviation, and the comparison between the two groups was carried out by using the two independent samples t-test. Non-normally distributed data were described as the median and the 25% and 75% quartiles, and the Mann-Whitney U test was performed. Pearson’s chi-squared test, Yate's correction for continuity or Fisher’s exact test was used for categorical variables analysis. The interobserver agreement of numeric and categorical variables was assessed using the intraclass correlation coefficient (ICC) and κ-statistic, respectively. The receiver operating characteristic (ROC) curve with the corresponding area under the curve (AUC) value was used to evaluate the discrimination ability of the prediction model in predicting VPI status in the training and validation set. The calibration curve and Hosmer-Lemeshow test were used to evaluate the goodness-of-fit of the prediction model, and a P-value of greater than 0.05 indicated a well goodness-of-fit. The decision curve analysis (DCA) was used to evaluate the clinical utility of the nomogram. Multivariate binary logistic regression, nomogram, validation, and calibration plots were done with the “rms” package of R software. The ROC was performed by the “pROC” package, and the DCA was performed with the function of “ggDCA”.

Additional file 6, Comparison of clinical and pathological data of clinical stage IA LUAD

| Clinical and pathological data | Training set (n = 283) | | |  | Internal validation set (n = 121) | | |  | External validation set (n = 81) | | |
| --- | --- | --- | --- | --- | --- | --- | --- | --- | --- | --- | --- |
|  | VPI-Negative ( n= 149) | VPI-Positive (n = 134) | *P*-value |  | VPI-Negative  (n = 61) | VPI-Positive  (n = 60) | *P*-value |  | VPI-Negative(n = 48) | VPI-Positive  (n = 33) | *P*-value |
| Gender |  |  | 0.137^b^ |  |  |  | 0.947^b^ |  |  |  | 0.117^b^ |
| Female | 92 (61.7%) | 71 (53.0%) |  |  | 40 (65.6%) | 39 (65.0%) |  |  | 33 (68.8%) | 17 (51.5%) |  |
| Male | 57 (38.3%) | 63 (47.0%) |  |  | 21 (34.4%) | 21 (35.0%) |  |  | 15 (31.3%) | 16 (48.5%) |  |
| Age | 59.0  (53.0, 66.0) | 61.0  (54.0, 67.0) | 0.180^a^ |  | 56.5 ± 9.3 | 59.1 ± 9.4 | 0.124^e^ |  | 60.3 ± 8.4 | 62.5 ± 8.9 | 0.265^e^ |
| Tumor location |  |  | 0.166^b^ |  |  |  | 0.807^b^ |  |  |  | 0.087^c^ |
| RUL | 46 (30.9%) | 40 (29.9%) |  |  | 20 (32.8%) | 16 (26.7%) |  |  | 22 (45.8%) | 13 (39.4%) |  |
| RML | 11 (7.4%) | 21 (15.7%) |  |  | 7 (11.5%) | 7 (11.7%) |  |  | 8 (16.7%) | 4 (12.1%) |  |
| RLL | 34 (22.8%) | 30 (22.4%) |  |  | 11 (18.0%) | 16 (26.7%) |  |  | 13 (27.1%) | 5 (15.2%) |  |
| LUL | 40 (26.8%) | 25 (18.7%) |  |  | 14 (23.0%) | 14 (23.3%) |  |  | 1 (2.1%) | 6 (18.2%) |  |
| LLL | 18 (12.1%) | 18 (13.4%) |  |  | 9 (14.8%) | 7 (11.7%) |  |  | 4 (8.3%) | 5 (15.2%) |  |
| Surgery type |  |  | 0.022^b^ |  |  |  | 0.237^b^ |  |  |  | 0.058^d^ |
| Sublobectomy | 48 (32.2%) | 27 (20.1%) |  |  | 19 (31.1%) | 13 (21.7%) |  |  | 7 (14.6%) | 0 (0.0%) |  |
| Lobectomy | 101 (67.8%) | 107 (79.9%) |  |  | 42 (68.9%) | 47 (78.3%) |  |  | 41 (85.4%) | 33 (100.0%) |  |
| Pathological grade |  |  | ＜0.001^d^ |  |  |  | 0.001^d^ |  |  |  | 0.693^c^ |
| MIA | 29 (19.5%) | 0 (0.0%) |  |  | 11 (18.0%) | 0 (0.0%) |  |  | 7 (14.6%) | 3 (9.1%) |  |
| IAC | 120 (80.5%) | 134 (100.0%) |  |  | 50 (82.0%) | 60 (100.0%) |  |  | 41 (85.4%) | 30 (90.9%) |  |

Note: ^a^:The Mann-Whitney U test. ^b^: Pearson’s chi-squared test. ^c^: Yate's correction for continuity. ^d^: Fisher’s exact test. ^e^: the two independent samples t-test.

VPI, visceral pleural invasion; RUL, Right upper lobe; RML, Right middle lobe; RLL, Right lower lobe; LUL, Left upper lobe; LLL, Left lower lobe; MIA, minimally invasive adenocarcinoma; IAC, invasive adenocarcinoma.

Additional file 7. Interobserver agreement analysis of CT features

| Qualitative indicators | Kappa value（95%CI） |  | Quantitative indicators | ICC（95%CI） |
| --- | --- | --- | --- | --- |
| Density type | 0.942 (0.907-0.977) |  | Tumor size | 0.954 (0.884-0.976) |
| Shape | 0.871 (0.822-0.920) |  | Solid component size | 0.955 (0.930-0.969) |
| Lobulation | 0.832 (0.724-0.940) |  | Pleural contact length | 0.989 (0.987-0.991) |
| Spiculation | 0.914 (0.869-0.959) |  | Solid pleural contact length | 0.996 (0.995-0.996) |
| Interface | 1.000 |  | DLP | 0.963 (0.956-0.970) |
| Air bronchogram | 0.917 (0.882-0.952) |  |  |  |
| Vacuole sign | 0.934 (0.899-0.969) |  |  |  |
| Cavity or cystic sign | 0.837 (0.747-0.927) |  |  |  |
| Vascular convergence sign | 0.910 (0.851-0.969) |  |  |  |
| Emphysema | 1.000 |  |  |  |
| Tumor-pleura relationship | 0.932 (0.907-0.957) |  |  |  |
| Solid attachment | 0.971 (0.949-0.993) |  |  |  |
| Pleural indentation | 0.968 (0.944-0.992) |  |  |  |

**Additional file 8.** Radiomics feature selection using the least absolute shrinkage (LASSO).

(Figure.ACEG) represents the selection of the optimal hyperparameter (λ) via 10-fold cross-validation of the least absolute shrinkage (LASSO) model based on minimum criteria for the GTV, GPTV_5_, GPTV_10_, and GPTV_15_ radiomics features, respectively. Binomial deviances from the LASSO regression cross-validation procedure are plotted as a function of log (λ).

(Figure.BDFG) represents the regression coefficients of LASSO, each colored line represents the variation curve of the feature coefficient with the log (λ). The black vertical line is drawn at the value selected using tenfold cross-validation in Fig.ACEG, respectively, the features with coefficients not equal to 0 were selected as the optimal feature subset to construct the radiomics model.

(AB) GTV model, (CD) GPTV_5_ model, (EF) GPTV_10_ model, (GH) GPTV_15_ model.

**
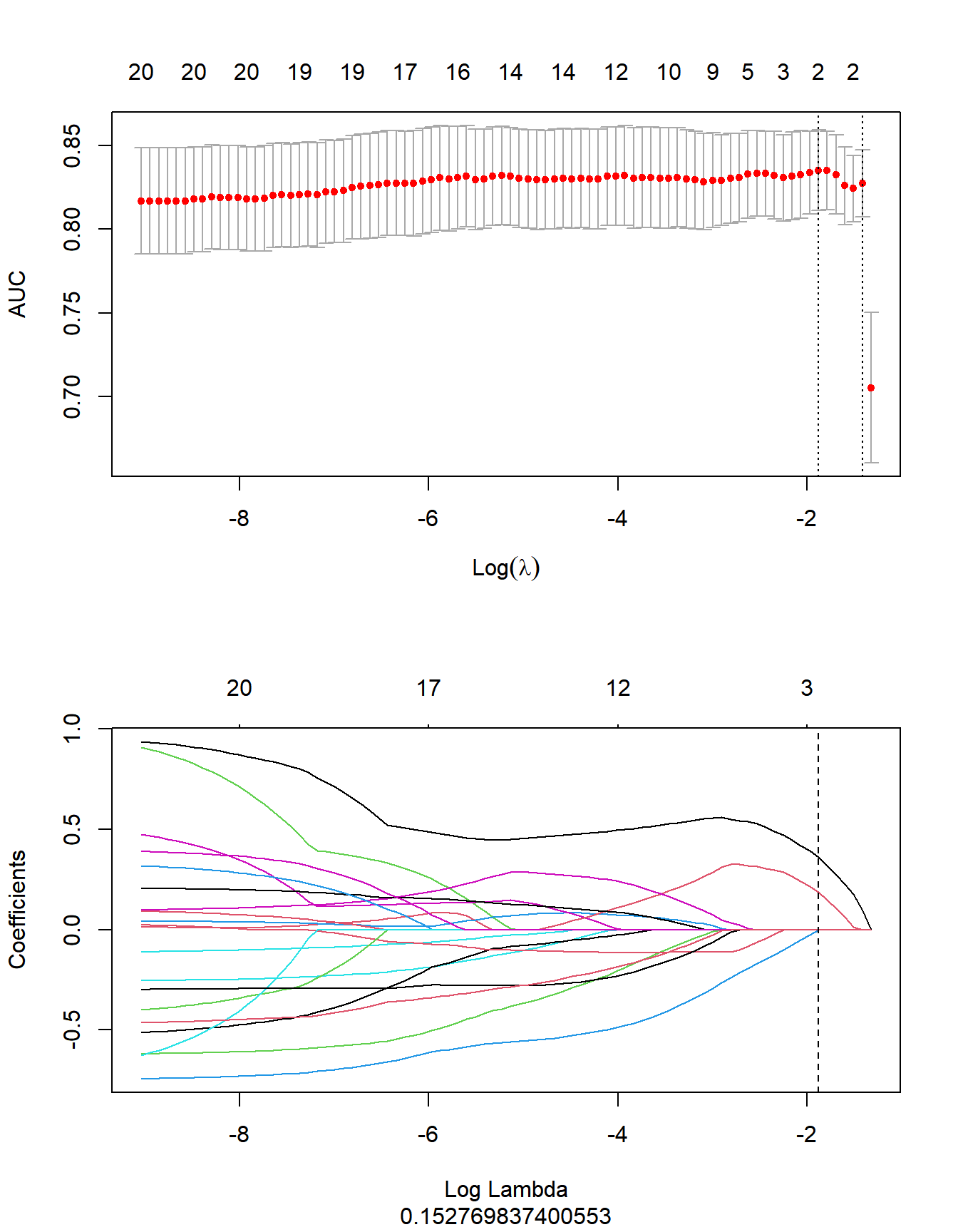

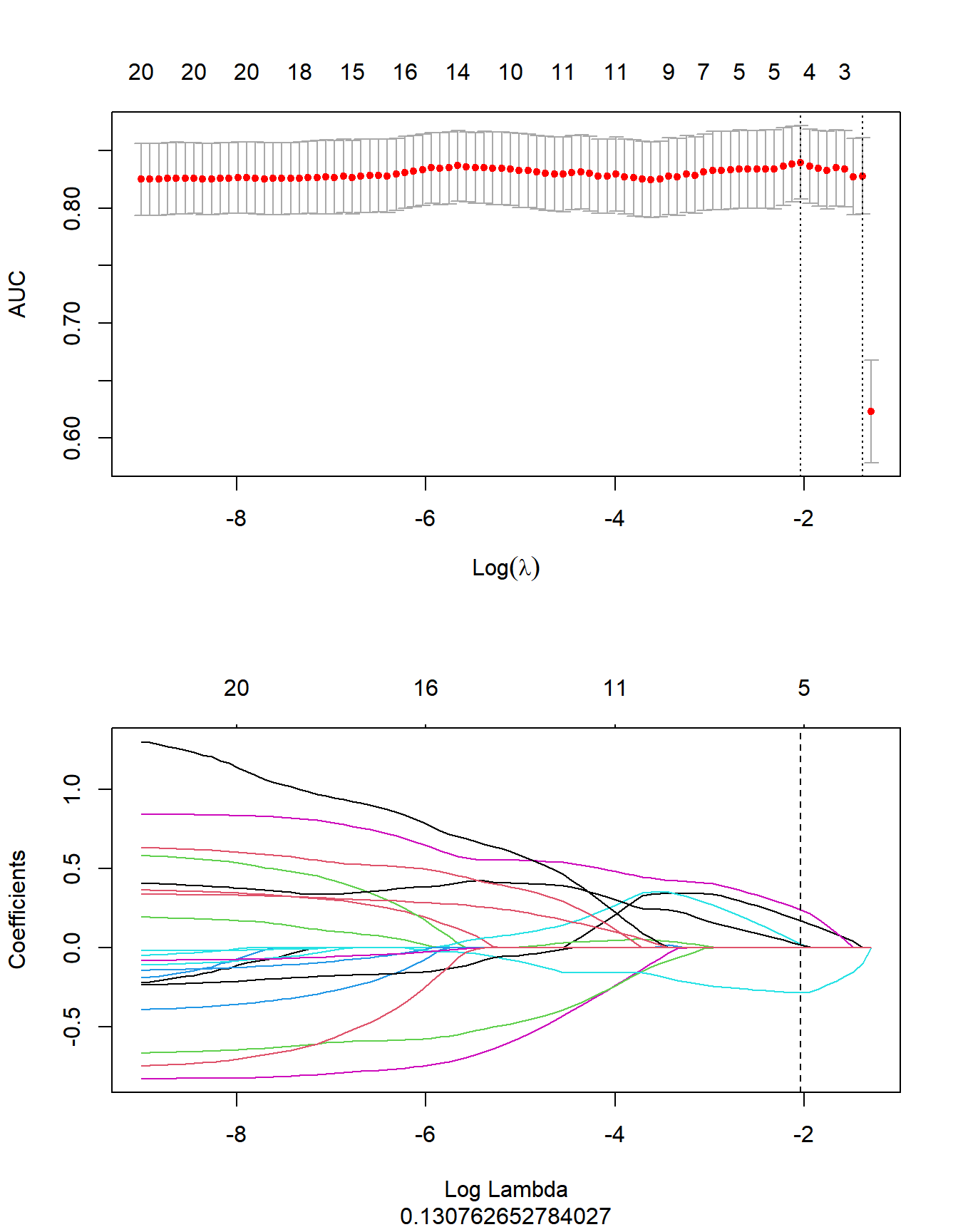
**

**C**

**A**

**B**

**D**

**
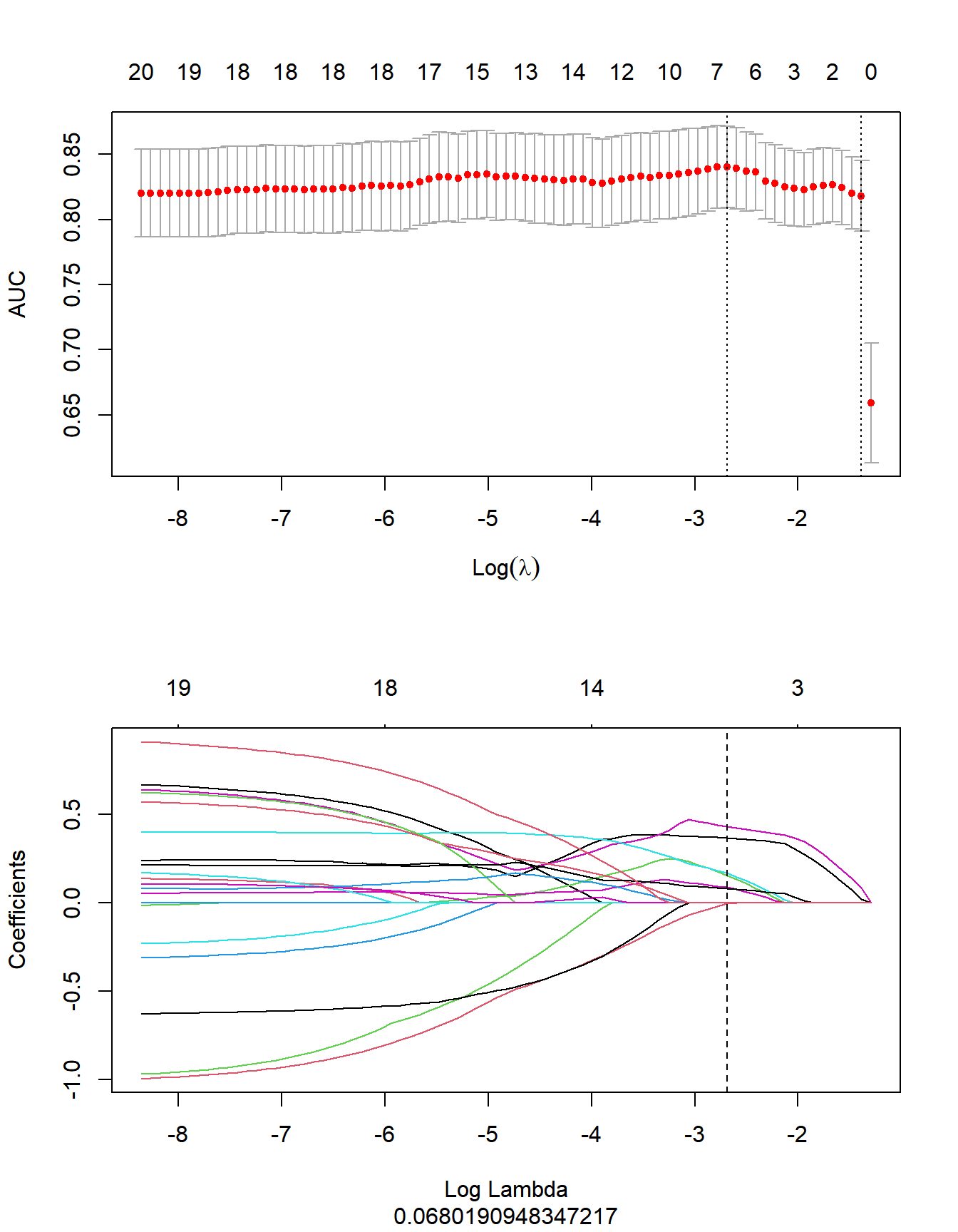

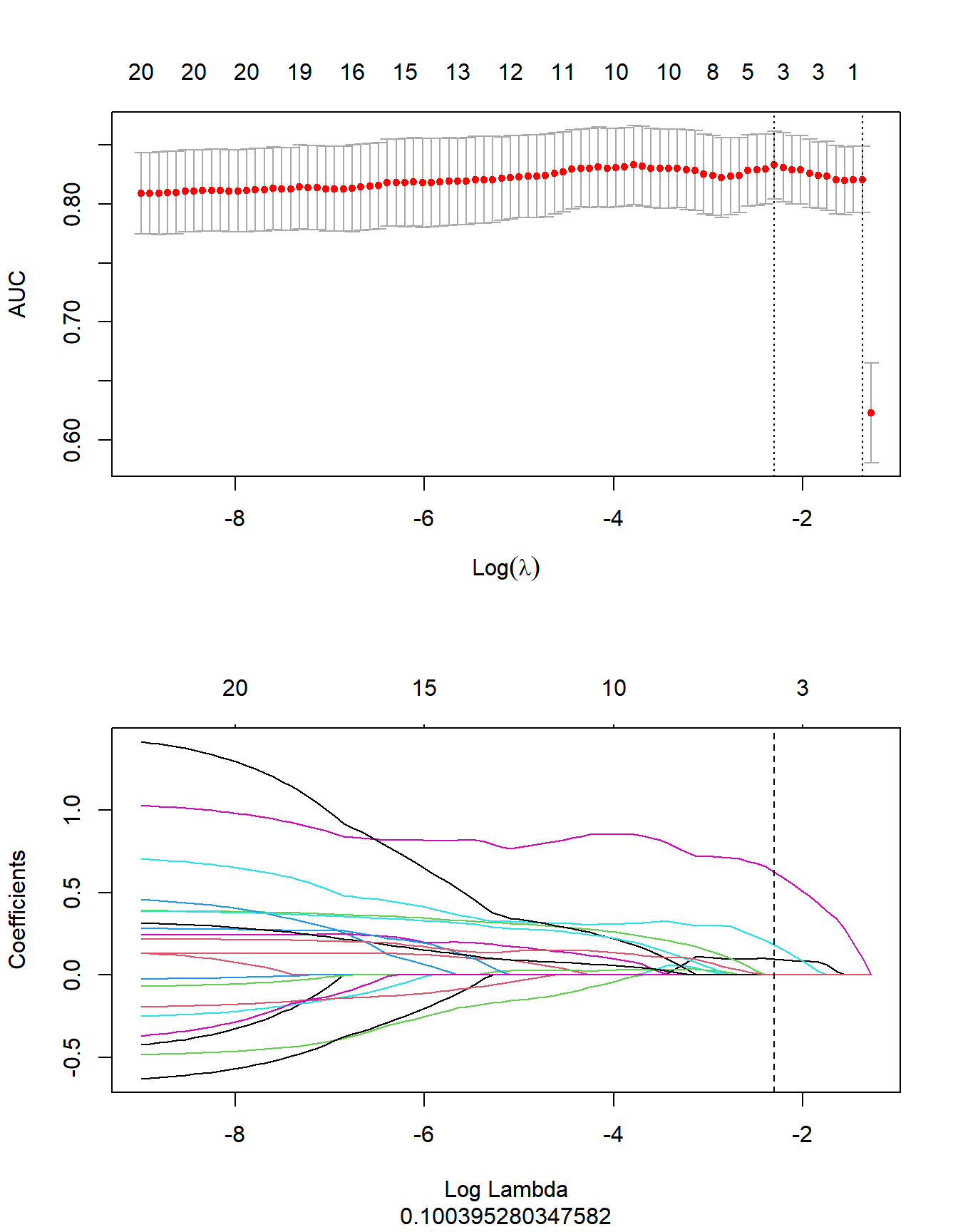
**

**E**

**G**

**F**

**H**

Additional file 9. ICCs of the selected features for GTV, GPTV_5_, GPTV_10_ and GPTV_15_ radiomics models

| Segmentation | Image type | Feature class | Feature name | ICC | |
| --- | --- | --- | --- | --- | --- |
|  |  |  |  | Interobserver | Intraobserver |
| GTV | LOG.2mm | GLSZM | Gray Level Non Uniformity | 0.847 | 0.965 |
|  | Wavelet.LLL | First Order | 90 Percentile | 0.949 | 0.954 |
| GPTV_5_ | Original | GLCM | Sum Squares | 0.956 | 0.965 |
|  | Wavelet.LLH | GLSZM | Zone Entropy | 0.980 | 0.979 |
|  | Wavelet.LLL | GLCM | Correlation | 0.986 | 0.989 |
|  | Original | GLSZM | Zone Entropy | 0.968 | 0.977 |
|  | LOG.2mm | First order | 10 Percentile | 0.840 | 0.868 |
| GPTV_10_ | Original | GLCM | Cluster Tendency | 0.973 | 0.978 |
|  | Wavelet.LLL | GLCM | Correlation | 0.990 | 0.990 |
|  | LOG.2mm | GLDM | Large Dependence Low Gray Level Emphasis | 0.943 | 0.956 |
|  | Wavelet.LLH | GLSZM | Zone Entropy | 0.993 | 0.992 |
|  | LOG.2mm | GLSZM | Zone Entropy | 0.982 | 0.983 |
|  | Original | GLSZM | Gray Level Variance | 0.939 | 0.972 |
|  | Original | GLSZM | Zone Entropy | 0.982 | 0.986 |
| GPTV_15_ | Original | GLCM | Cluster Shade | 0.978 | 0.983 |
|  | Wavelet.LLL | GLCM | Correlation | 0.993 | 0.993 |
|  | Original | GLSZM | Gray Level Variance | 0.948 | 0.977 |

**Additional file 10.** Radscore formula of GTV, GPTV_5_, GPTV_10_ and GPTV_15_ radiomics models

GTV-Radscore = 0.362 * log_sigma_2_0_mm_3D_glszm_GrayLevelNonUniformity + 0.187 * wavelet_LLL_firstorder_90Percentile + -0.108

GPTV_5_-Radscore = 0.169 * original_glcm_SumSquares + 0.022 * wavelet_LLH_glszm_ZoneEntropy + 0.017 * wavelet_LLL_glcm_Correlation + 0.24 * original_glszm_ZoneEntropy + -0.285 * log_sigma_2_0_mm_3D_firstorder_10Percentile + -0.115

GPTV_10_-Radscore = 0.365 * original_glcm_ClusterTendency + 0.428 * wavelet_LLL_glcm_Correlation + -0.005 * log_sigma_2_0_mm_3D_gldm_LargeDependenceLowGrayLevelEmphasis + 0.153 * wavelet_LLH_glszm_ZoneEntropy + 0.166 * log_sigma_2_0_mm_3D_glszm_ZoneEntropy + 0.086 * original_glszm_GrayLevelVariance + 0.08 * original_glszm_ZoneEntropy + -0.126

GPTV_15_-Radscore = 0.096 * original_glcm_ClusterShade + 0.625 * wavelet_LLL_glcm_Correlation + 0.18 * original_glszm_GrayLevelVariance + -0.12

**Additional file 11.** Box-type scatter plot of Radscore predicting VPI in patients.

The abscissa label 0 represents VPI-negative, label 0 represents VPI-positive, and the ordinate represents Radscore. (A) GTV model, (B) GPTV_5_ model, (C) GPTV_10_ model, (D) GPTV_15_ model

**A**


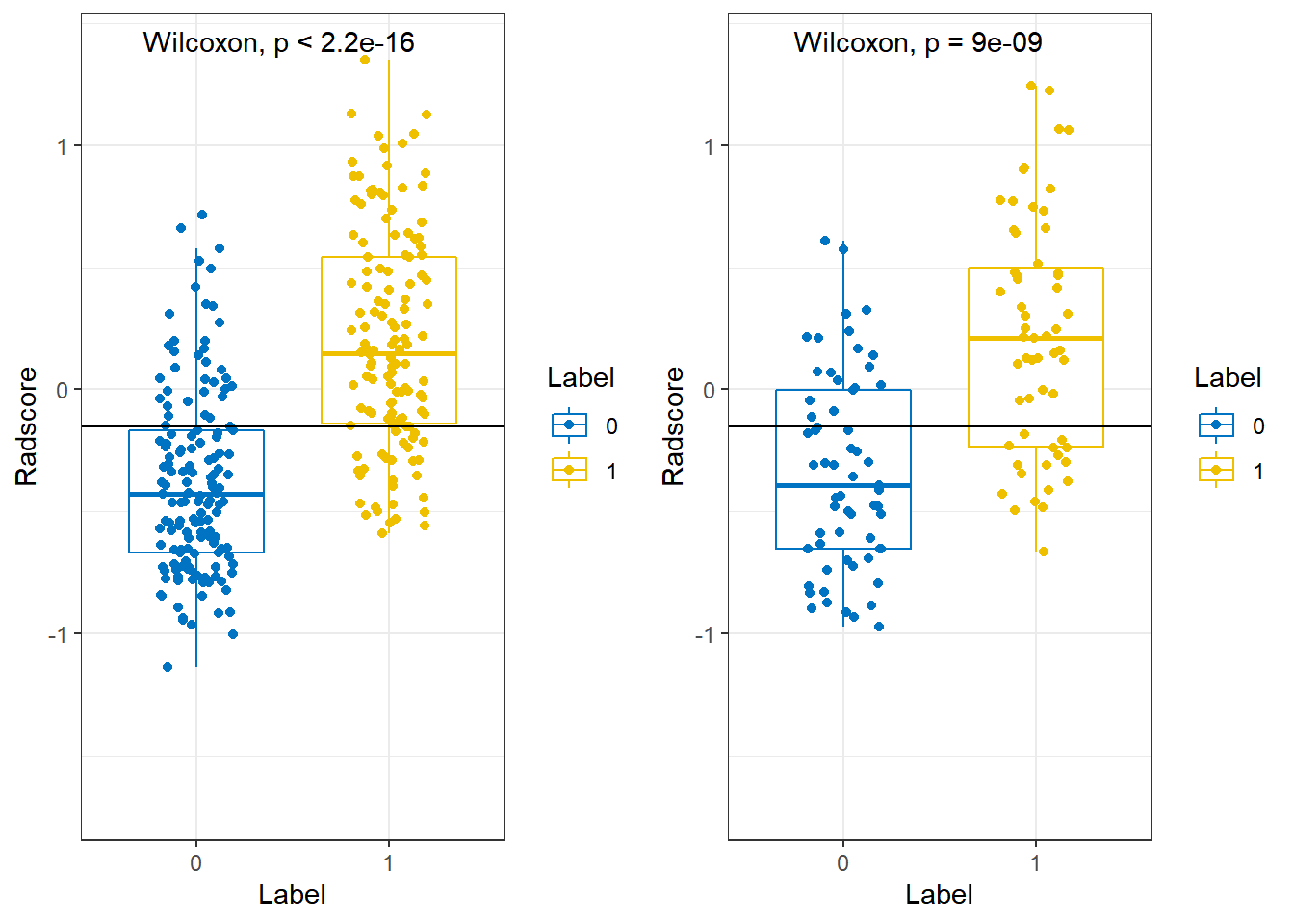

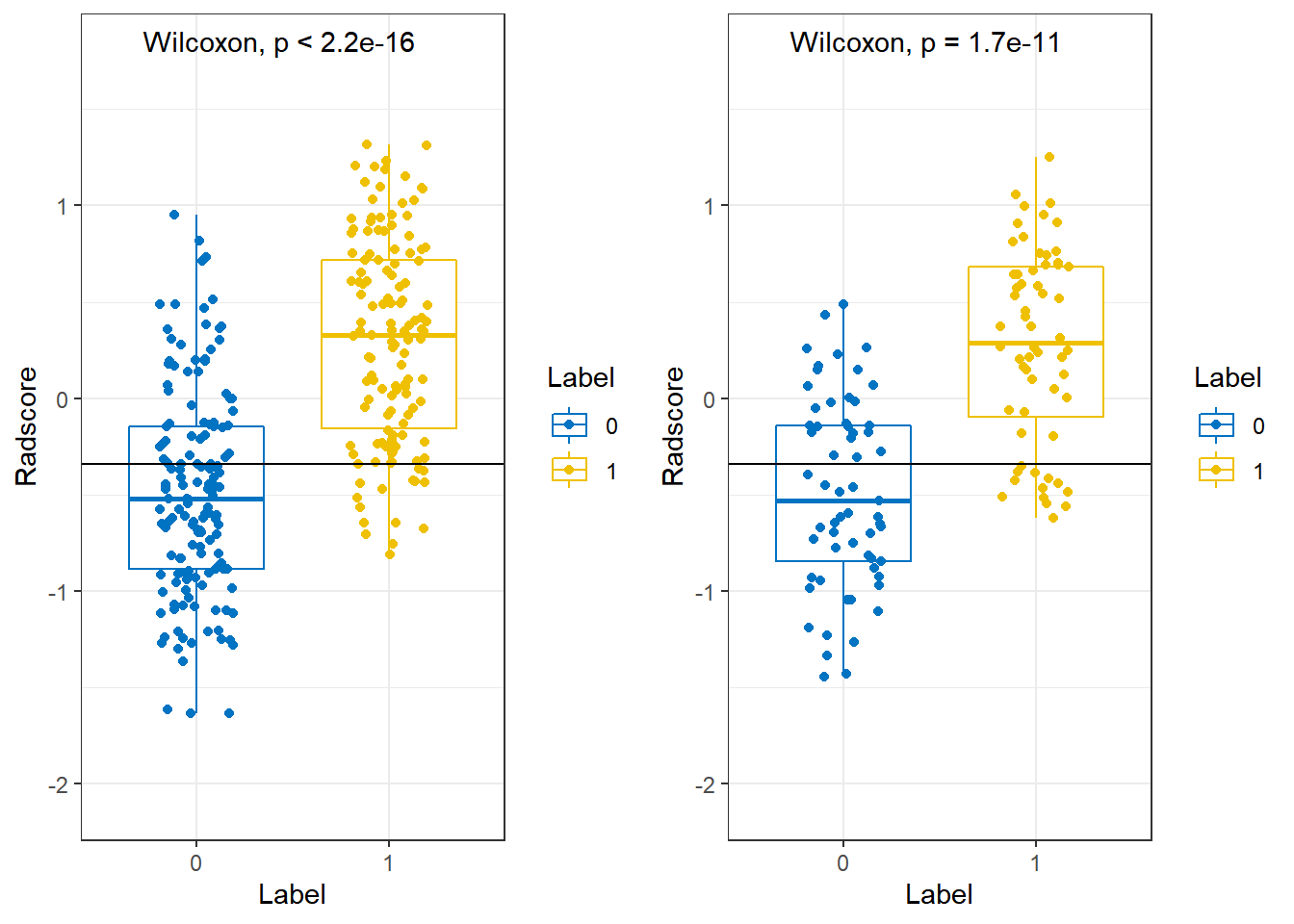


**C**

**B**


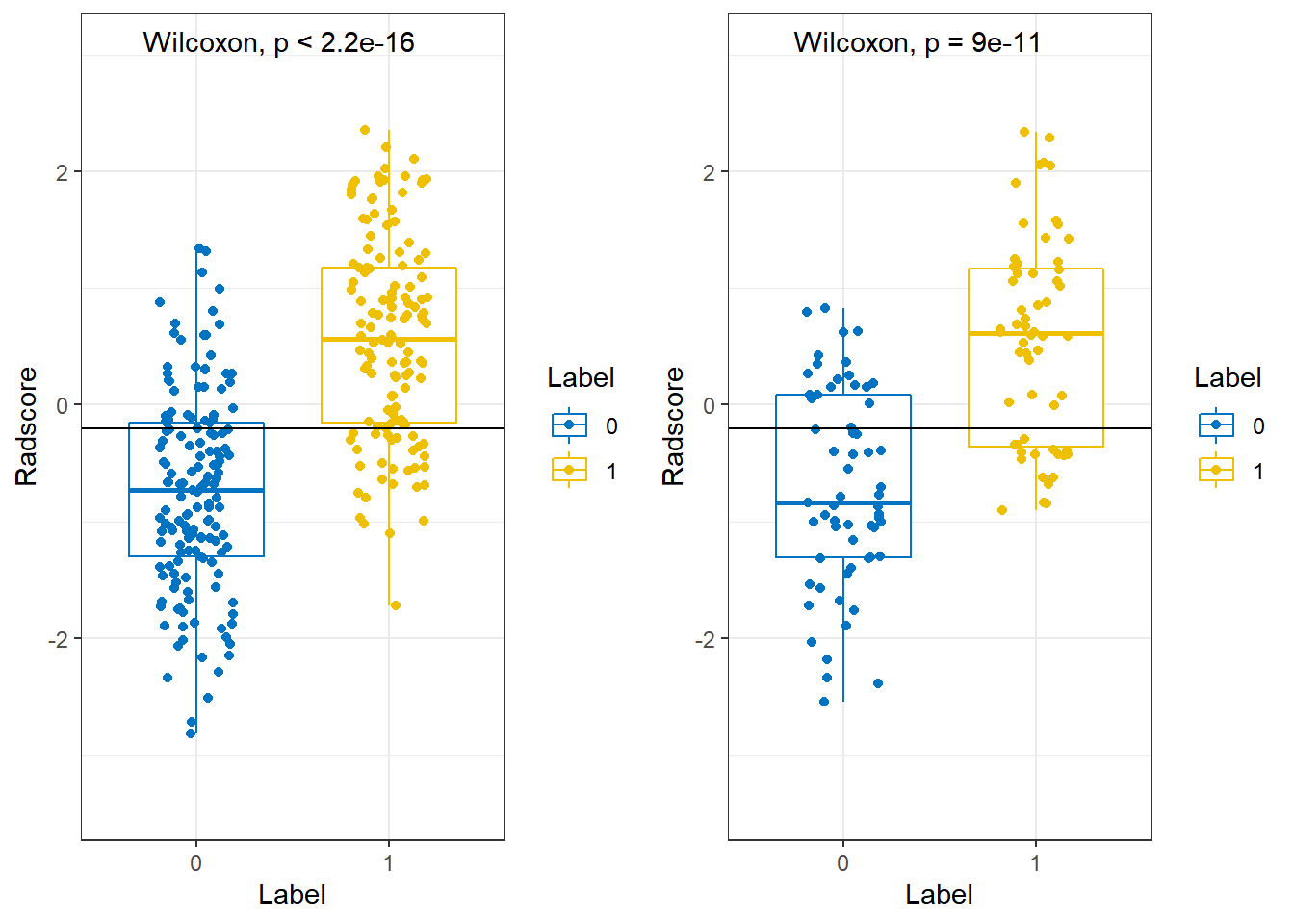

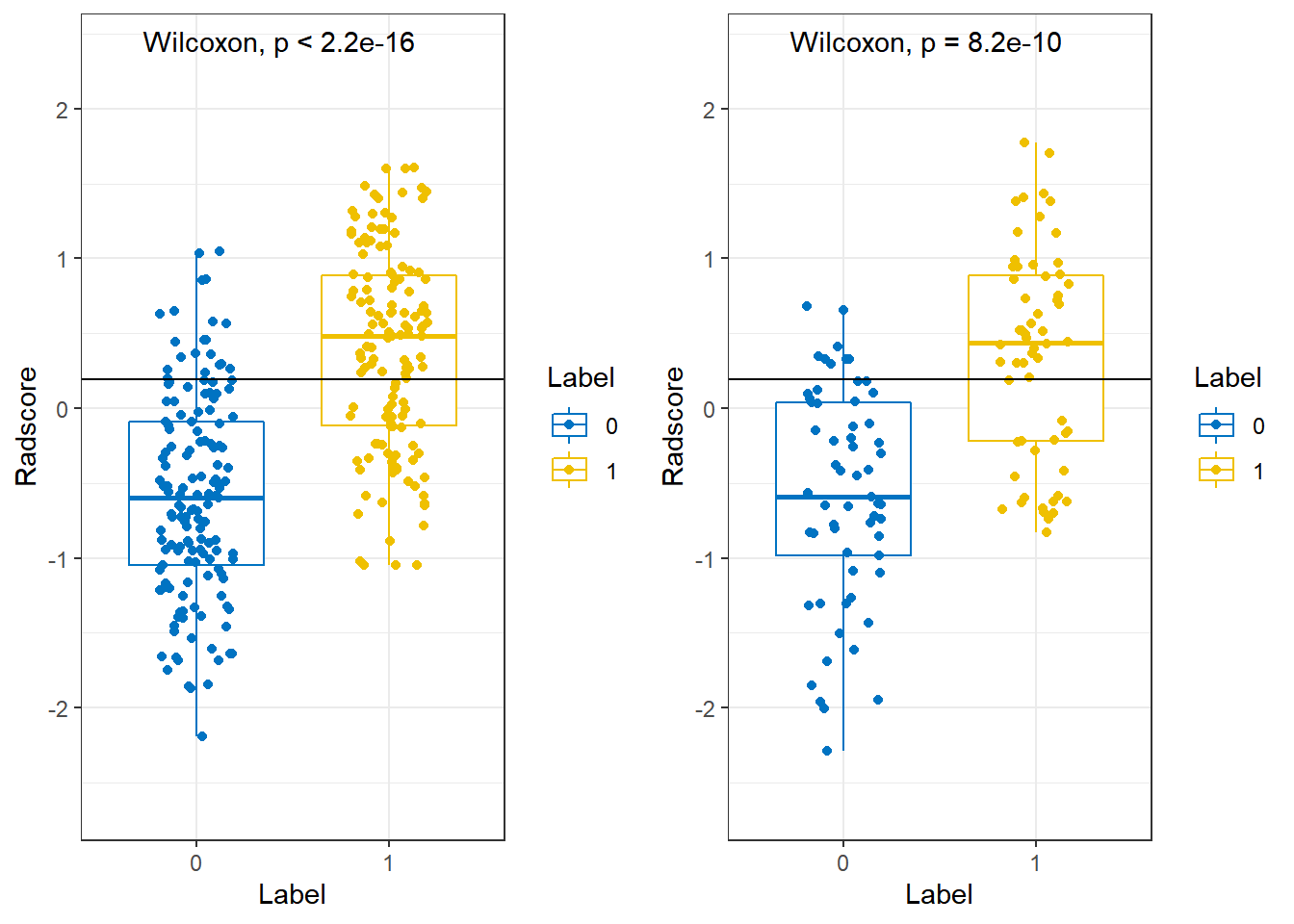


**D**

**Additional file 12.** ROC curves of each radiomics model in the three sets.

(A) Training set, (B) Internal validation set, (C) External validation set

**A**

**B**


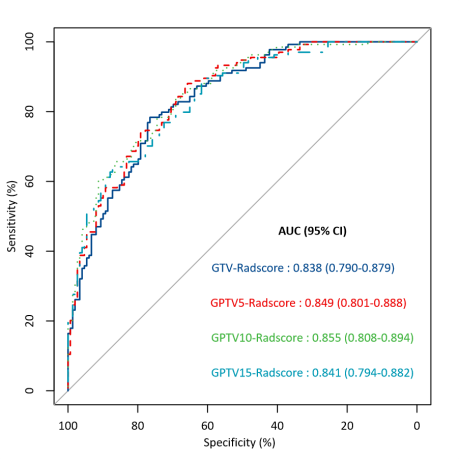

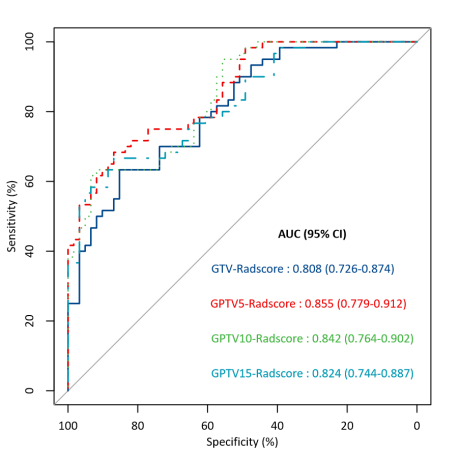


**C**


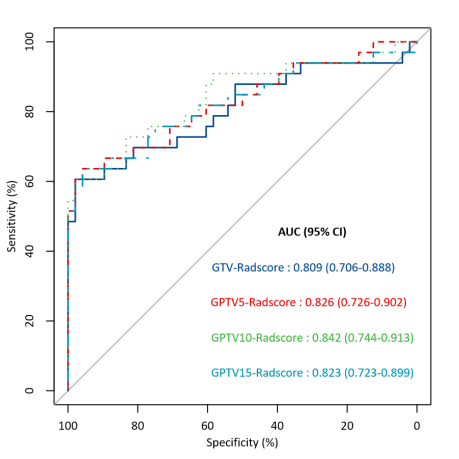

Supplement: Supplementary file 1 — Supplementary Material 1 [file 13019_2024_2807_MOESM1_ESM.doc]
